# Endothelial glycocalyx sensitivity to chemical and mechanical sub-endothelial substrate properties

**DOI:** 10.3389/fbioe.2023.1250348

**Published:** 2023-10-30

**Authors:** Mohammad Hamrangsekachaee, Ke Wen, Narges Yazdani, Rebecca K. Willits, Sidi A. Bencherif, Eno E. Ebong

**Affiliations:** ^1^ Chemical Engineering Department, Northeastern University, Boston, MA, United States; ^2^ Bioengineering Department, Northeastern University, Boston, MA, United States; ^3^ Laboratoire de BioMécanique et BioIngénierie (BMBI), UMR CNRS, Sorbonne Universités, Université de Technologie of Compiègne (UTC), Compiègne, France; ^4^ Harvard John A. Paulson School of Engineering and Applied Sciences, Harvard University, Cambridge, MA, United States; ^5^ Neuroscience Department, Albert Einstein College of Medicine, New York, NY, United States

**Keywords:** endothelial cells, substrate stiffness, gelatin-based hydrogel, mechanotransduction, glycocalyx

## Abstract

Glycocalyx (GCX) is a carbohydrate-rich structure that coats the surface of endothelial cells (ECs) and lines the blood vessel lumen. Mechanical perturbations in the vascular environment, such as blood vessel stiffness, can be transduced and sent to ECs through mechanosensors such as GCX. Adverse stiffness alters GCX-mediated mechanotransduction and leads to EC dysfunction and eventually atherosclerotic cardiovascular diseases. To understand GCX-regulated mechanotransduction events, an *in vitro* model emulating *in vivo* vessel conditions is needed. To this end, we investigated the impact of matrix chemical and mechanical properties on GCX expression via fabricating a tunable non-swelling matrix based on the collagen-derived polypeptide, gelatin. To study the effect of matrix composition, we conducted a comparative analysis of GCX expression using different concentrations (60–25,000 μg/mL) of gelatin and gelatin methacrylate (GelMA) in comparison to fibronectin (60 μg/mL), a standard coating material for GCX-related studies. Using immunocytochemistry analysis, we showed for the first time that different substrate compositions and concentrations altered the overall GCX expression on human umbilical vein ECs (HUVECs). Subsequently, GelMA hydrogels were fabricated with stiffnesses of 2.5 and 5 kPa, representing healthy vessel tissues, and 10 kPa, corresponding to diseased vessel tissues. Immunocytochemistry analysis showed that on hydrogels with different levels of stiffness, the GCX expression in HUVECs remained unchanged, while its major polysaccharide components exhibited dysregulation in distinct patterns. For example, there was a significant decrease in heparan sulfate expression on pathological substrates (10 kPa), while sialic acid expression increased with increased matrix stiffness. This study suggests the specific mechanisms through which GCX may influence ECs in modulating barrier function, immune cell adhesion, and mechanotransduction function under distinct chemical and mechanical conditions of both healthy and diseased substrates.

## 1 Introduction

Atherosclerosis, the underlying cause of cardiovascular diseases, accounts for 37% of deaths in individuals up to 70 years old (Virani et al., 2021). Atherosclerosis is a medical condition that involves the narrowing of the vessel lumen, leading to an increase in flow resistance, as well as the thickening and hardening of the vessel wall (Laroia et al., 2003; Kohn et al., 2015). Severe atherosclerosis is usually associated with increased blood vessel rigidity that occurs with age and in cases of hypertension (Tegos et al., 2001; Benetos et al., 2002; Mitchell et al., 2010).

The endothelium lining the vessel luminal surface is critical to vascular health. Endothelial cells (ECs) play a crucial role in regulating various cardiovascular functions, such as vessel tone regulation, selective permeability, hemostasis, and mechanotransduction. EC dysfunction is widely suggested as a primary contributor to the development of atherosclerosis (Deanfield et al., 2007; Suowen et al., 2021). Since ECs are exposed to different mechanical stimuli at the interfaces between ECs and blood flow, as well as between ECs and underlying vessel tissues (Tarbell and Pahakis, 2006; Jansen et al., 2017), numerous studies have attributed EC dysfunction with abnormal alterations in mechanical cues within the vascular environment. These cues include fluid shear stress derived from blood and tissue stiffness (Topper et al., 1996; Bonetti et al., 2003; Mitchell et al., 2010). Considering the attention given to the impact of shear stress on cellular responses, herein the focus is on stiffness. In healthy blood vessels, the substrate stiffness underlining ECs typically ranges from 2.5 to 5 kPa. This range may vary based on the measuring methods, including non-invasive techniques like pulse wave velocity (PWV), ultrasound, and magnetic resonance imaging (MRI) for *in vivo* and *ex vivo* atomic force microscopy (AFM) (Engler et al., 2004; Klein et al., 2009; Peloquin et al., 2011; Stroka and Aranda-Espinoza, 2011; Lee et al., 2017). In pathological conditions, the substrate undergoes remodeling, and becomes stiffened (stiffness >10 kPa) (Peloquin et al., 2011). These changes typically arise due to abnormal alterations in substrate composition. For instance, the loss of collagen, whose primary role is to provide the main tensile strength of the artery wall, can elevate the risks associated with foam-cell macrophage activation (Newby, 2008). In order to reduce the mortality associated with atherosclerosis, it is crucial to elucidate the underlying factors and mechanistic causes of EC dysfunction that originate from the substrate.

One of the key regulators of EC function is glycocalyx (GCX), which is a multifunctional layer that covers vascular ECs. GCX primarily consists of proteoglycans, glycoproteins, and glycosaminoglycan (GAG) chains. Proteoglycans are present as core proteins bound to the cell membrane with GAGs attached to them (Tarbell and Cancel, 2016; Zeng et al., 2018). Studying GCX has been challenging due to its complex and delicate structure; however, the evidence demonstrating its influence on EC function is steadily increasing. As a mechanotransducer, GCX has been extensively reported for its ability to sense fluid shear stress within its surrounding microenvironment. This, in turn, mediates flow-induced activation of endothelial NO synthase (eNOS), as well as the expression of adhesion molecules and dysregulation of inflammatory genes (Ebong et al., 2010; Yen et al., 2015; Hamrangsekachaee et al., 2022). In contrast to the shear stress, the impact of the substrate matrix, especially the solid-derived forces originating from it, has received less attention in research. Recent studies by Tarbell’s group, our esteemed GCX research collaborators, have provided intriguing evidence regarding the effect of substrate stiffness on GCX expression (Mahmoud et al., 2021a; Mahmoud et al., 2021b). It is incumbent upon us to take the baton and continue working to enhance our understanding of GCX mechano-response to substrate stiffness. Moreover, the potential effect of substrate chemistry on GCX expression remains unknown. Therefore, further research is necessary to elucidate the role of mechanical and chemical properties of the substrate matrix on GCX expression and, subsequently, on EC function.

This paper first aimed to investigate GCX chemical sensitivity to the substrate matrix material. Gelatin methacrylate (GelMA) derived from collagen was synthesized as a physiologically relevant matrix. GelMA was chosen as the material of preference due to its advantageous characteristics. These include its high biocompatibility, the presence of adhesive molecules like arginine-glycine-aspartic acid (RGD), and the ability to fine-tune its mechanical properties. Additionally, GelMA allows for easy and cost-effective hydrogel fabrication, thanks to its versatility in crosslinking methods, such as light-based techniques and redox-induced polymerization. Furthermore, GelMA exhibits the capability to encapsulate various types of cells and bioactive molecules. This versatility makes it a material of choice for the subsequent development of the model, facilitating the inclusion of vascular smooth muscle cells (VSMCs) to enrich the model’s complexity and physiological relevance (Van Den Bulcke et al., 2000; Yue et al., 2015; Lavrentieva et al., 2020; Cuvellier et al., 2021).

Another objective was to investigate GCX chemical sensitivity to different substrate coatings. It is worth noting that fibronectin is commonly used as a substrate coating in atherosclerosis studies to improve cell attachment (Stroka and Aranda-Espinoza, 2011; Yeh et al., 2012; Mahmoud et al., 2021a; Mahmoud et al., 2021b). In fact, our group has completed many informative studies using fibronectin as a substrate for cultured ECs (Ebong et al., 2011; Ebong et al., 2014; Mensah et al., 2017; Harding et al., 2018; Mensah et al., 2020). However, excessive deposition of fibronectin by ECs has been observed under adverse shear stress conditions in animal models of cardiovascular disease. The deposition occurs at early stages of atherosclerosis, preceding deposition of fibrinogen which typically occurs later (Hahn et al., 2009; Hamrangsekachaee et al., 2022). Moreover, Wayne Orr et al. demonstrated that fibronectin coating, compared to collagen I coating, upregulates atherogenic genes. Notably, they observed increased expression levels of intercellular adhesion molecule 1 (ICAM-1), vascular cell adhesion molecule 1 (VCAM-1), and Nuclear factor-κB (NF-κB) (Orr et al., 2005; Chirinos, 2012). Therefore, in the present study it was hypothesized that the composition of the substrate coating could potentially contribute to dysregulation in GCX expression. To test this hypothesis, first glass slides were coated with fibronectin, gelatin, and GelMA at a concentration of 60 μg/mL (this is considered as baseline concentration, or 1x concentrated). In light of the fact that the hydrogels would be composed of GelMA and given that GelMA could be tuned to vary the concentration of adhesive RGD molecules, experiments were also performed to further assess GCX sensitivity to different concentrations (greater than 60 μg/mL) of GelMA coating. For the various coating conditions examined, GCX expression was evaluated using wheat germ agglutinin (WGA) staining.

The last but primary objective of this paper was to investigate GCX mechanical sensitivity to substrate stiffness. GelMA matrix was used as previously mentioned. Furthermore, on transitioning from glass to matrix it was confirmed whether GelMA alone was sufficient to support cell attachment and growth along with GCX expression, or whether gelatin coating on the GelMA was required for enhancement of cell attachment. Once the GelMA coating material was defined, GelMA hydrogels with stiffnesses of 2.5 and 5 kPa (representing a healthy matrix), and 10 kPa (representing a diseased matrix) were chosen (Engler et al., 2004; Klein et al., 2009; Peloquin et al., 2011; Stroka and Aranda-Espinoza, 2011). It was hypothesized that 10 kPa hydrogels downregulate GCX expression compared to 2.5 and 5 kPa hydrogels. In addition to investigating the expression of GCX using WGA, major polysaccharide components of the GCX, such as heparan sulfate (HS), sialic acid (SA), and hyaluronic acid (HA), were also examined.

## 2 Materials and methods

### 2.1 Materials

Gelatin Type A (300 bloom) from porcine skin, methacrylic anhydride (MAH), (trimethoxysilyl)propyl methacrylate, tetramethylethylenediamine (TEMED), ammonium persulfate (APS), and HA binding protein (HABP, 385,911)—the binding of which to HA was demonstrated by Heinegård et al. and Fuhrmann et al. (Heinegård and Hascall, 1974; Fuhrmann et al., 2015)—as well as goat serum, were procured from Sigma-Aldrich (St. Louis, MO). Phosphate-buffered saline (PBS) and human recombinant fibronectin were acquired from Gibco (Waltham, MA). Bovine serum albumin (BSA), 5/6-carboxyfluorescein succinimidyl ester (NHS-FITC), acetone, glacial acetic acid, deuterium oxide (D2O), paraformaldehyde, and AF488-conjugated goat anti-mouse IgM (SA5-10294) were obtained from Thermo Fisher Scientific (Waltham, MA). Human umbilical vein endothelial cells (HUVEC), vascular basal medium, and EC growth kit were sourced from the American Type Culture Collection (ATCC, Manassas, VA). Biotinylated WGA lectin (PL-1025), the binding of which to GCX was validated by Gabor et al. (Gabor et al., 1997), biotinylated elderberry bark lectin (B-1305), whose binding to SA was confirmed by Ishigaki et al. (Ishigaki and Itoh, 2022), and 4’,6-diamidino-2-phenylindole (DAPI)-containing mounting media were purchased from Vector Laboratories (Burlingame, CA). The HS antibody (clone F58-10E4), the binding of which to HS was validated by David et al. (David et al., 1992), was acquired from Amsbio LLC (Cambridge, MA). AF488-conjugated streptavidin (AB_2337249) was purchased from Jackson ImmunoResearch Inc. (West Grove, PA).

### 2.2 Synthesis of GelMA

To improve our understanding of EC function pertinent to atherosclerotic disease, we first need to advance our available *in vitro* models to more accurately mimic healthy and diseased conditions. The specific interest of the present study is to understand the impact of substrate matrix mechanical properties on GCX expression on ECs, requiring the fabrication of a reproduceable, mechanically, and chemically tunable EC-compatible hydrogel. To fulfill this requirement, GelMA synthesis was carried out. First, type A, 300 bloom gelatin from porcine skin was dissolved in 0.25M carbonate-bicarbonate buffer at a concentration of 10% (w/v) at 55°C. The pH of the gelatin solution was adjusted to 9.5 using 0.1M NaOH. MAH was added to the solution in a volume ratio of 1:100 to start the methacrylation reaction. The solution was stirred at 500 RPM for an hour to complete the reaction (Zhu et al., 2019). The resulting GelMA solution was then added dropwise to an excess acetone solution at 200 RPM to cause precipitation (Kim et al., 2014). The product was collected on absorbent papers, dried in a vacuum oven at room temperature (RT), and stored at −20°C until further use. To synthesize FITC-labeled gelatin or GelMA, the previously established protocol was followed (Rezaeeyazdi et al., 2018). Briefly, gelatin or GelMA was dissolved in a sodium bicarbonate solution at a concentration of 10% (w/v), and the pH was adjusted to 8.5. NHS-FITC was added to the solution at a concentration of 0.01% (w/v), and the mixture was stirred at 500 RPM overnight. FITC-labeled gelatin GelMA was obtained by precipitating the solution in excess acetone and then dried in a vacuum oven at RT.

### 2.3 Chemical characterization of GelMA

GelMA was characterized to evaluate the degree of substitution. Therefore, proton nuclear magnetic resonance (^1^H NMR) spectra were obtained using Varian Inova-500 NMR and Brucker 500 MHz NMR spectrometers. GelMA was dissolved in D_2_O at a concentration of 2 mg/mL and the spectrum was acquired at RT, with a 15 Hz sample spinning at a 45^o^ angle and an 8 μs delay for 128 scans. The methacryloyl peak areas were integrated at 5.4 and 5.6 ppm. The degree of substitution was determined by calculating the ratio of the number of amine groups to the gelatin amines prior to the methacrylation reaction. The aromatic region was used as a control for the concentration of gelatin and GelMA (Rezaeeyazdi et al., 2018).

To assess the consumption of methacrylate groups during polymerization, the hydrogels were washed, lyophilized for 3 days, snap frozen, crushed, and suspended in D_2_O for ^1^H NMR. The disappearance of vinylic peaks was used as evidence for the consumption of the methacryloyl residues.

### 2.4 Glass coating

To enhance the handling and fabrication of hydrogels, plain microscope slides (1 mm thickness) were precisely cut into 2.5 × 2.5 cm^2^ pieces using a FlipScribe glass cutter (LatticeGear). The cut slides were thoroughly washed with soap, sonicated for 5 min in ethanol, and air-dried. Subsequently, the slides were functionalized with 3-(Trimethoxysilyl)propyl methacrylate to improve their surface properties and allow the covalent attachment of hydrogels during the polymerization process. A 0.5% solution of 3-(Trimethoxysilyl)propyl methacrylate in absolute ethanol was prepared, and 3% dilute glacial acetic acid solution (10% solution in deionized (DI) water) was added to the mixture. The resulting solution was poured over the glass slides and allowed to react for approximately 5 min on a rocker (80 RPM). After completion of the reaction, the slides were washed with ethanol 2–3 times and stored in the dark at RT.

### 2.5 Hydrogel preparation

Hydrogels were synthesized using a redox-induced free radical polymerization method. GelMA was dissolved in DI water at various concentrations and heated to 45°C. TEMED (12 mM for 2.5 kPa hydrogel, 24 mM for 5 kPa hydrogel, and 6.25 mM for 10 kPa hydrogel) and APS (12 mM for 2.5 kPa hydrogel, 24 mM for 5 kPa hydrogel, and 24.5 mM for 10 kPa hydrogel) were added to the GelMA solution and mixed thoroughly. The resulting solution was quickly poured into Teflon molds at RT and allowed to cross-link. After completion of the reaction, the hydrogels were transferred to 1x PBS and equilibrated at 37°C to remove the remaining reactants and byproducts.

### 2.6 Mechanical compression and rheological testing

To select the substrates emulating the physiological and pathological matrix stiffnesses, the mechanical tests were conducted using a TA Electroforce 5,500 mechanical loading device (TA Instruments, New Castle, United States) with a calibrated 1,000 lb load cell at RT in an aqueous environment. Cylindrical samples with dimensions of 8 × 4 cm^2^ were inserted between the compression plates. The compression plate was lowered at a rate of 0.01 mm/s until a total displacement of 50% of the sample height was achieved. Data for load and displacement were recorded and used to calculate stress and strain. The slope of the stress-strain curve between 5% and 10% strain was used to determine the Young’s modulus.

The polymerization time and rheology of GelMA hydrogels were evaluated using an ARES RFS 3 rheometer with stainless-steel cone and plate (cone angle of 0.0403 rad, gap size of 0.0508 mm, 25 mm). To determine the linear viscoelastic region of the hydrogel, a strain sweep test was performed at a frequency of 10 rad/s with a strain range of 0.1%–100%. The linear viscoelastic region was determined by plotting the storage modulus (G′) against shear strain (γ%) on a log-log plot and identifying the point at which G′ exhibited strain-dependent behavior. A time sweep test was carried out at a frequency of 10 rad/s and a strain of 3% to determine the gelation point, which was reported when G′ and G″ intersected at a phase angle of 45°, indicating the onset of gel formation.

### 2.7 Swelling behavior of hydrogels

The swelling behavior of the hydrogels was quantified by calculating the ratio of the weight of the swollen hydrogel to the weight of the dehydrated gel after 48 h of lyophilization. Additionally, to assess the dimensional changes resulting from swelling up to equilibrium, the hydrogels were incubated perpendicularly in PBS at 37°C overnight. Microscopy images of the hydrogels were captured using an AXIO observer Z1 microscope (Cal Zeiss Meditec AG), and their thicknesses were measured.

### 2.8 Endothelial cell culture

To investigate the effect of substrate chemical and mechanical properties on GCX expression, HUVEC were used. The HUVECs were cultured in vascular cell basal medium supplied with EC growth kit containing various growth factors: recombinant human vascular endothelial growth factor (rh-VEGF: 5 ng/mL), epidermal growth factor (rh-EGF: 5 ng/mL), basic fibroblast growth factor (rh-FGF basic: 5 ng/mL), insulin-like growth factor-1 (rh-IGF-1:15 ng/mL), L-glutamine (10 mM), heparan sulfate (0.75 Units/mL), hydrocortisone (1 μg/mL), ascorbic acid (50 μg/mL), fetal bovine serum (2% FBS), and 1% penicillin/streptomycin. The cells were incubated in a humidified incubator at 37°C with 5% CO_2_. For the experiments, passage 4 to 7 HUVECs were used, consistent with our previously published studies (Mensah et al., 2020).

To study the effect of composition on GCX expression, No. 1.5 microscope cover glasses were prepared for the experiments. The cover glasses were sterilized either by autoclaving or by treating them with 70% ethanol, and then washed with PBS. The coating materials were prepared at an initial concentration of 60 μg/mL, referred to as 1x. Human fibronectin was used as the control at 1x concentration. Gelatin and GelMA were used at different concentrations: 1x, 5x, 10x, 100x, and 400x. 400x is the highest practical concentration close to that of GelMA solution used for fabricating hydrogels with a stiffness of 2.5 kPa. The coating solutions were applied to cover glasses and incubated at 37°C for 45 min. Afterward, the cover glasses were rinsed with PBS. HUVECs were seeded on the coated surfaces at a density of 5,000 cell/cm^2^. The media was changed every other day until the HUVECs reached full confluency. Prior to the experimental endpoint, 0.5% BSA was added to media overnight to enhance GCX stability.

To investigate the effect of stiffness, hydrogels were prepared as described in Section 2.4 and cut into 2.5 × 2.5 × 0.1 cm^3^ dimensions. The hydrogels were then incubated in PBS at 37°C overnight. Subsequently, the hydrogels were sterilized using 70% ethanol on a rocker for 45 min. Following sterilization, the hydrogels were treated with 1x gelatin for 45 min at 37°C and incubated in cell culture media overnight prior to cell seeding. HUVECs showed a lower cell attachment to the hydrogels in comparison to the glass slides. Therefore, ECs were seeded on the hydrogels at a higher density of 100,000 cell/cm^2^, and the media was changed every 2 days until the cell layer reached 100% confluency occurring after 3–4 days. To stabilize GCX, 0.5% BSA was added to media overnight before fixation.

### 2.9 Histological analysis

To study GCX response to substrate matrix conditions, the GCX and its major polysaccharide components, including heparan sulfate (HS), hyaluronic acid (HA), and sialic acid (SA) were labeled. Once the monolayer of ECs reached 100% confluency (after 3–4 days), a wash with 1% BSA was performed, followed by fixation. A fixative solution containing 2% paraformaldehyde and 0.5% glutaraldehyde in PBS was used for 30 min to prepare the samples for histology. For saccharide, a fixative solution of 4% paraformaldehyde was used for 15 min. The fixation was done at RT before applying blocking agents (BSA or goat serum) for 1 h. Lectins were used to bind to sugar moieties for labeling GCX and SA. The samples were incubated with biotinylated WGA and biotinylated elderberry bark lectin, respectively, at a dilution of 1:100 each for 1 hour at RT. Secondary labeling was carried out by incubating the samples with Alexa flour 488 (AF488)-conjugated streptavidin at a dilution of 1:1,000, for 1 hour at RT. For HS and HA labeling, the samples were transferred to humid chambers and incubated with clone F58-10E4 antibody against HS and biotinylated HA binding protein, respectively, at a dilution of 1:100 each for 3 days at 4°C. Secondary labeling for HS and HA was performed by incubating the samples with AF488-conjugated goat anti-mouse IgM at a dilution of 1:400 and AF488-conjugated streptavidin at a dilution 1:50, respectively, for 1 h. Finally, the samples were rinsed and mounted using DAPI-containing mounting media. Negative control studies (Supplementary Figure S1) were performed to assess the lectin, binding protein, and antibody specificity and extract non-specific staining data to be subtracted from histology data of GCX sensitivity to substrate chemistry and stiffness.

### 2.10 Imaging and image analysis

Z-stack images were captured using Zeiss LSM 800 and 710 confocal microscopes (Cal Zeiss Meditec AG) at ×63 (oil emission) magnification. Further imaging parameters are detailed in Supplementary Table S1. GCX expression on HUVECs, including both GCX and its components, was quantified using multiple methods: 1) normalized GCX thickness, measured in the orthogonal direction; 2) normalized GCX component expression, determined by normalizing mean fluorescence intensity (MFI) measurements, which indicate aggregate density, in the *en face* view; and 3) percent GCX coverage of the EC apical surface, also determined in the *en face* view. These methods are based on well-established GCX analysis approaches. This being said, it is understood in the GCX research community that measured GCX thickness, expression, and coverage values may be influenced by various factors, including microscope resolution, occasional internalization of labeling agents, and fusion of apical and basal signals. Consequently, in this paper, GCX measurements are not always shown as absolute but may be normalized to show relative GCX differences when comparing the impact of various conditions on the GCX.

For GCX thickness quantification, a custom Python program was developed. The thickness of GCX in the X-Z dimension was estimated from the GFP (AF488) channel, which captured the GCX intensity at a wavelength of 488 nm. To reduce image noise and refine the details, a Gaussian blur filter was first applied to the sample image. Then, the image was thresholded with the Otsu method, which divides the pixels into two classes based on intensity histogram and separates the foreground GFP (AF488) fluorescence regions of interest (ROI) from the background. The algorithm proceeded with randomly drawing a vertical line within the ROI along the *X*-axis and counting the number of pixels (or z-stack layers) that had fluorescence intensity greater than the threshold on the line. Multiplying this count by the length of pixels (or the intervals between z-stack layers) provided the ROI thickness, which represented the GCX thickness. The process was repeated for a total of 50 lines, and the average of their thickness values was calculated as the average GCX thickness expressed on the sample image. A detailed description of the thickness quantification performed using Python is provided in the images and caption of Supplementary Figure S2.

For quantifying the normalized GCX component expression, a different Python algorithm was developed. This algorithm measured average GFP (AF488) intensity expressed in every pixel of the image in the *en face* view, which represented the sample Mean Fluorescence Intensity (MFI). MFI was taken as an indicator of the aggregate density of the expressed GCX components that were under examination. The data then were normalized with respect to a reference group in each figure by dividing the intensity values by the mean intensity of the reference group.

To quantify the percentage of GCX coverage of the EC apical surface area, indicating the distribution of GCX components, CellProfiler 4.2.4 (Broad Institute, MA) was utilized. However, for this particular study, only the quantification of HS, SA, and HA is reported. Measurements of percent area covered by GCX (WGA-labeled GCX) on ECs were obtained but are not included in this report due to the observation of a nearly complete area coverage of GCX across all the samples.

All custom-designed Python modules used for analyzing the captured confocal images in both the orthogonal and *en face* dimensions, as well as for quantifying the GCX, are publicly available online at https://github.com/KE-Chloe-WEN/GCX_quant.

### 2.11 Statistical analysis

In the context of this study, the term “N" is used to denote biological replicates, which refers to an independent set of experiments conducted to validate the results and ensure the reliability of the findings. On the other hand, “n" represents operational replicates, which entails repetitions of specific treatments or interventions within each experimental group, aimed at assessing the consistency and reproducibility of the treatment effects. Power analysis was performed to confirm the statistical rigor of the experimental design **(** Supplementary Figure S3 **)**. All data are reported as mean ± standard error of the mean (SEM). Statistical analyses were conducted using Minitab software, and the generated plots were created using GraphPad Prism software. To determine significant differences related to various factors with a level of significance of *α* = 0.05, ANOVA with Tukey’s HSD (honestly significant difference) *post hoc* test was employed for multiple comparisons. In the figures, the significance levels and corresponding *p*-values are indicated as follows: non-significant (ns), *p < 0.05, **p < 0.01, ***p < 0.001, and ****p < 0.0001.

## 3 Results

### 3.1 Synthesis and characterization of GelMA

**FIGURE 1:**
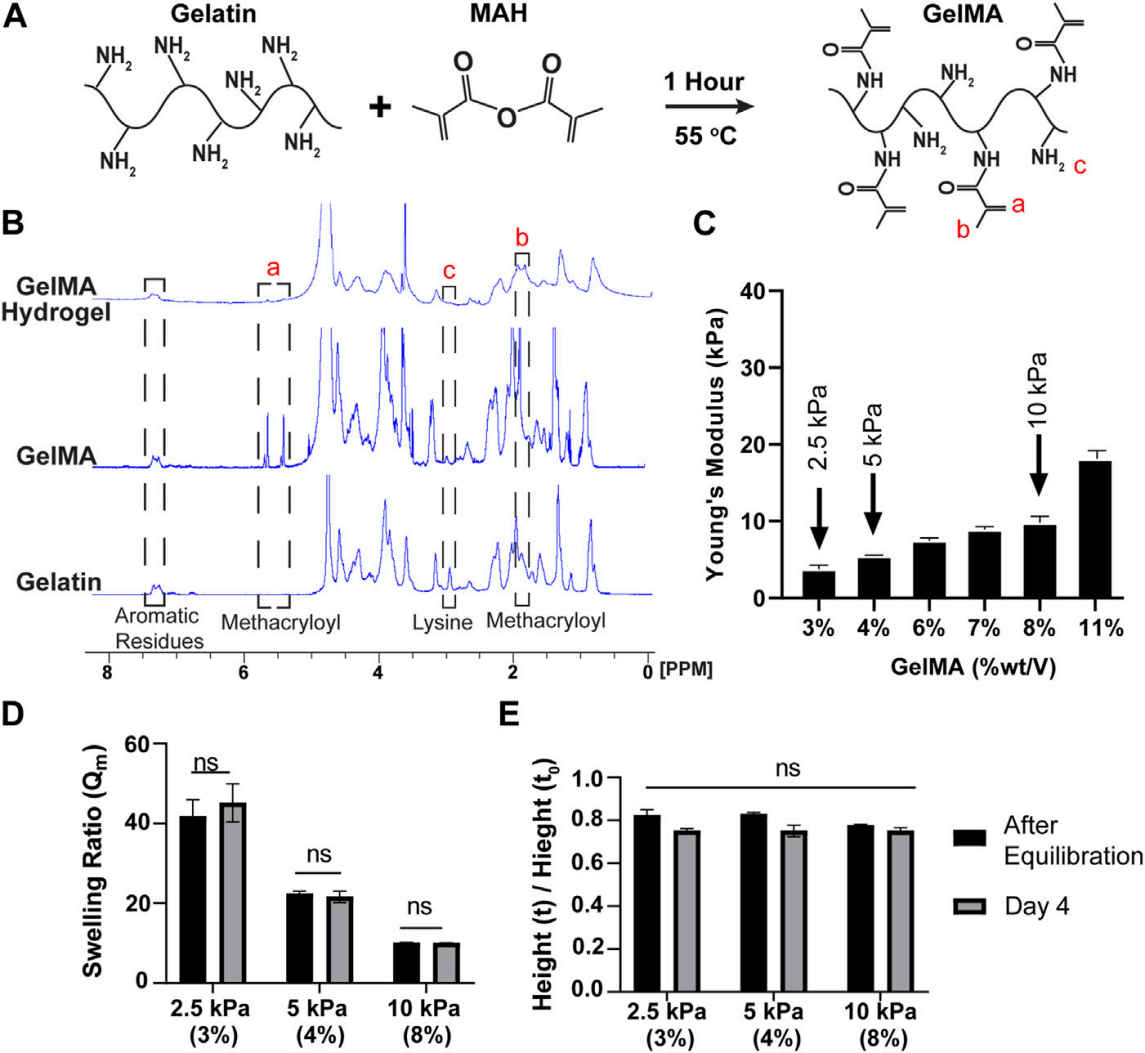
GelMA synthesis via substitution of lysine residues result in non-swelling hydrogels with tunable stiffnesses. **(A)** Schematic illustration of GelMA fabrication through a reaction between gelatin and MAH. **(B)** ^1^H-NMR spectra for gelatin, GelMA pre-polymerization, and GelMA post-polymerization. Refer to dashed lined boxes for relevant peaks. Methacryloyl peaks (i.e., vinylic and methyl groups) appeared after modification of gelatin; while lysine group was used during substitution reaction (Krishnamoorthy et al., 2019). Subsequently vinylic peaks disappeared after crosslinking due to their consumption during polymerization. Aromatic peaks were stable in all stages and can be used as a reference. **(C)** The mechanical properties of the hydrogels were evaluated through a compression test to select those with the desired stiffness. Three% and 4 % (w/v) GelMA hydrogels are considered as physiological substrates and 10 % (w/v) were considered as pathological substrate (mean ± SEM). **(D, E)** Two methods for swelling behavior evaluation. **(D)** The weight of the gels before and after drying, demonstrating the weight of the gels remained stable over time (mean ± SEM). **(E)** The height measurements of the gels confirmed the data obtained from the part B showing non-swelling behavior of the gels (mean ± SEM).

Figure 1A illustrates the chemical synthesis of GelMA. ^1^H NMR spectra were utilized to determine the presence of Methacryloyl moieties following the chemical reactions. In Figure 1B, the appearance of new peaks at 1.9, 5.4, and 5.6 ppm in GelMA confirmed successful methacrylation. Specifically, the chemical shifts around 5.4–5.6 ppm indicated the presence of vinylic protons, while the emergence of a peak at 1.9 ppm served as a marker for the methyl group (Kim et al., 2014; Rezaeeyazdi et al., 2018; Zhu et al., 2019). Furthermore, the absence of the peak at 3 ppm, compared to gelatin, confirmed that the lysine residues were successfully substituted with methacrylate groups during the chemical reaction. The degree of methacrylation was estimated to be approximately 80%. NMR spectra also confirmed that the methacrylate groups were consumed during the polymerization process, as evidenced by the disappearance of the vinylic peaks (Rezaeeyazdi et al., 2018).

### 3.2 Mechanical properties of hydrogels

The stiffness of GelMA hydrogels was characterized via a compression test. As illustrated in Figure 1C, the compression moduli increased with the concentration of GelMA. Lower concentrations of GelMA resulted in the formation of softer hydrogels capable of withstanding large deformations, whereas higher polymer concentrations yielded harder hydrogels with increased stiffness. To mimic physiological and pathological substrate matrix stiffnesses, hydrogels with stiffnesses of 2.5, 5, and 10 kPa were selected for further experiments (Charbonier et al., 2019). These corresponded to GelMA concentrations of 3, 4, and 8% (wt/vol), respectively. To investigate the gelation times, oscillatory time sweep experiments were conducted on GelMA hydrogels with concentrations of 3, 4, and 8% (wt/vol), as shown in Supplementary Figure S4. Prior to the test, TEMED and APS were mixed with the polymer solutions, and the resulting mixture was loaded into the gap between the cone and plate. Initially, G′ values were smaller than G″ values, indicating that the crosslinks were insufficient to transform the liquid samples into a solid state. However, as the crosslinked network increased over time, the elastic behavior of the hydrogels became more prominent, and the G’ values surpassed the G” values. The point at which the two moduli crossed over was designated as the gelation time, indicating the dominance of the elastic behavior in the gels. The G′ values exhibited an exponential increase and eventually reached a plateau, while G″ slightly decreased. As anticipated, the gelation process occurred faster for samples with higher polymer concentrations. The gelation times for the 3, 4, and 8% GelMA samples were approximately 132, 78, and 48 s), respectively. These times included the 30 s required for the pre-initiation process before initiating the test. Furthermore, the value of tan(δ) decreased over time and was approximately 1 at the crossover point. As the concentration of GelMA increases, a decrease in gelation time was observed. This effect can be attributed to the reduced spatial distance between GelMA molecules, which increases the likelihood of collision for methacrylate groups. As a result, the crosslinking process occurs more rapidly, resulting in a shorter gelation time.

### 3.3 Swelling behavior of hydrogels

The swelling behavior of hydrogels is an important property to consider in biomedical applications. Specifically, hydrogels used for *in vitro* studies of vascular ECs should exhibit minimal swelling to uphold a consistent flow regime and shear stress on the ECs on the gel are inserted into a flow chamber environment (this is beyond the scope of this paper and the focus of our ongoing work that will be reported in future publications). In this study, we investigated the influence of GelMA concentration and incubation time in PBS on the swelling ratio. As depicted in Figure 1D, an increase in GelMA concentration resulted in a decrease in the swelling ratio. This phenomenon can be attributed to the physical interlock and chemical binding of the polymer network, which impede the hydrogel’s ability to swell. Meanwhile, the denominator of the Q_m_ equation (Q_m_ = hydrated weight/dry weight) increased due to a higher proportion of the solid phase remaining after lyophilization. The swelling ratios for the 2.5, 5, and 10 kPa hydrogels were measured as 41.7, 22.4, and 10.1, respectively. It is worth mentioning that the hydrogels, although they swell, eventually stabilize and reach an equilibrium point after 4 hours of incubation in PBS at 37°C. There was no significant difference between the samples at two different time points: the time of gel equilibration and day 4 after equilibration. As shown in Figure 1E, we also assessed the dimensional changes in the hydrogels by measuring the height alteration. This was done by dividing the height of the hydrogel at equilibrium with the initial height of the sample immediately after polymerization. It was observed that there was no swelling and a non-significant shrinkage in the samples, which could be attributed to the highly cross-linked nature of the hydrogels.

### 3.4 The effect of substrate composition on GCX

GCX sensitivity to the substrate was first assessed by examining the influence of three substrate materials, fibronectin, gelatin, and unpolymerized GelMA, which were initially used as coatings on glass slides to eliminate the potential mechanical influence that hydrogel would have on cell behavior. Fibronectin coating was included in this work because it is commonly employed as a coating component in EC studies for atherosclerosis. However, its deposition occurs during the diseased phase when ECs gain an inflammatory phenotype (Hahn et al., 2009; Hamrangsekachaee et al., 2022) and, therefore, the use of fibronectin may interfere with the natural behavior of cells in response to their environment. Gelatin and GelMA coatings were both used because they are derived from collagen, which is physiologically relevant. GelMA is considered to be the most ideal coating because it can be extended from a coating format to a gel format easily and relatively inexpensively, and in a manner that will permit substrate cell and bioactive molecule encapsulation. GelMA can also be tuned to increase the concentration of adhesive RGD molecules, and better support cell adhesion and growth. It was unclear whether and how the coatings made of fibronectin, gelatin, and GelMA, of variable concentrations, would affect the expression of GCX.

**FIGURE 2:**
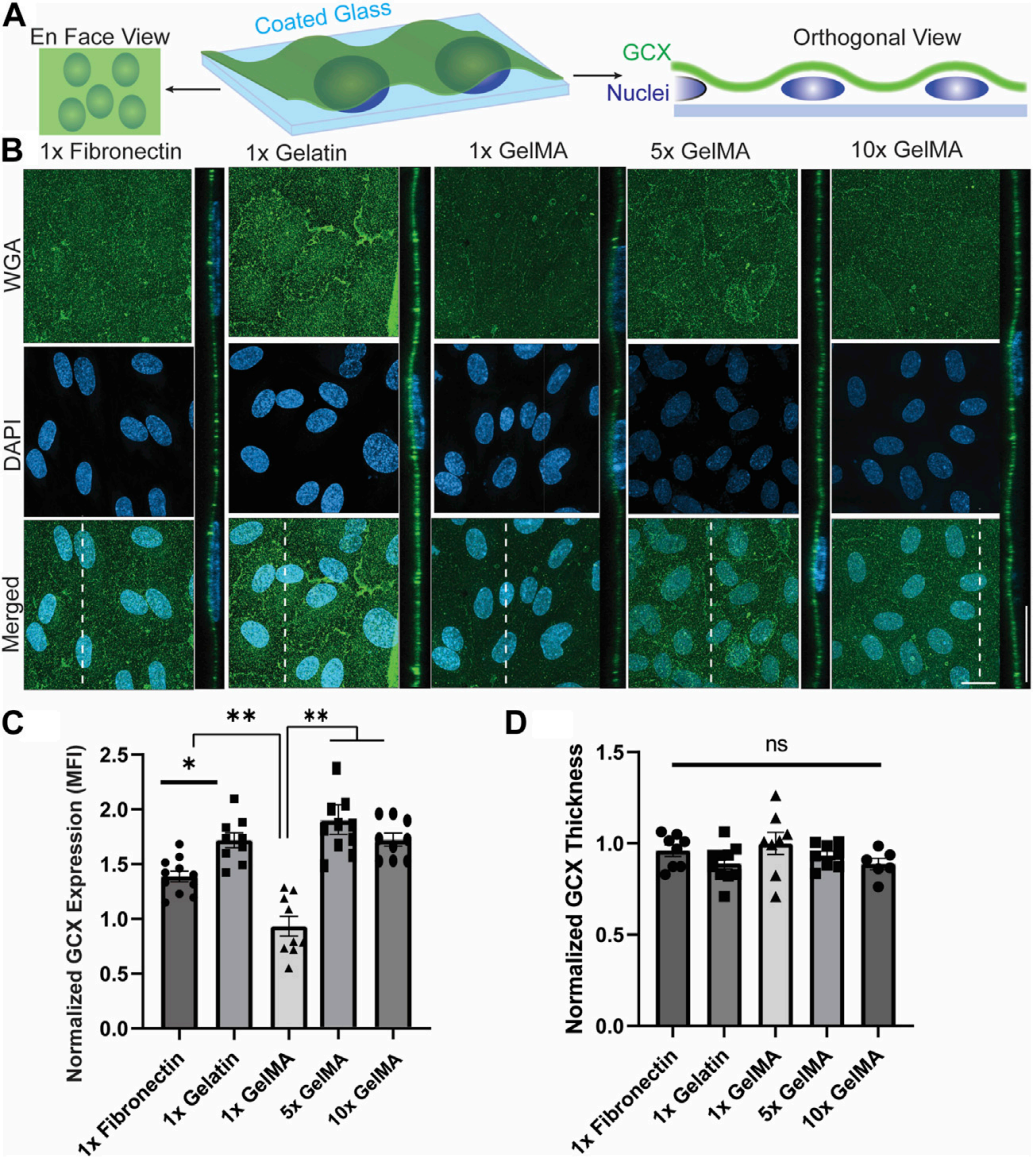
The composition and concentration of coatings regulate GCX expression on glass slides. **(A)** Schematic illustration of the *en face* and orthogonal views used for analyzing the GCX. Dashed lines correspond to positions of orthogonal views. **(B)** *En face* and orthogonal views showing effect of 1x fibronectin, 1x gelatin, 1x GelMA, 5x GelMA, *versus* 10x GelMA substrate material on apical GCX expression. Green is WGA, the marker of whole GCX, and blue is DAPI, the EC nuclei marker (scale bars are 20 μm). **(C)** The MFI from *en face* views of microscopic images showing that substrate material did indeed affect GCX expression, with gelatin significantly increasing expression while GelMA significantly decreasing expression, both compared to fibronectin. Additionally, increasing the concentration of GelMA enhanced the GCX MFI to levels comparable with GCX expression in the gelatin group. This demonstrates that the concentration of GelMA peptides in the substrate could alter GCX expression (N = 3, n = 3, mean ± SEM, *p < 0.05, **p < 0.01). **(D)** Thickness of the GCX was measured from the orthogonal view and did not show any significant difference between the coating materials and concentrations (N = 3, *n* = 3, mean ± SEM). The non-normalized data for this plot are as follows: 1x Fibronectin, 1.23 μm ± 0.040; 1x Gelatin, 1.14 μm ± 0.044; 1x GelMA, 1.28 μm ± 0.078; 5x GelMA, 1.20 μm ± 0.030; and 10x GelMA, 1.14 μm ± 0.040.

To address this question, whole GCX (WGA) was examined, and the normalized GCX expression (MFI) from the *en face* view of microscopic images was used to evaluate the effect of substrate material on apical GCX expression (Figure 2A). The orthogonal views of z-stack images were used to measure the thickness of the GCX (Figure 2A). The results showed that the substrate material had a significant effect on whole GCX (WGA) expression. Specifically, gelatin significantly increased expression by more than 30%, while 1x GelMA significantly decreased expression by about 30%, compared to fibronectin (Figures 2B, C). While overall expression responded to differences in substrate composition (fibronectin *versus* gelatin *versus* 1x GelMA), the thickness of the whole GCX (WGA) did not change substantially in response to substrate composition although there was a trend indicating that the measured thickness increased when GCX expression decreased (Figure 2D). For GelMA, GCX expression was further assessed by conducting a dose-response study of the effect of increasing GelMA concentration while controlling for stiffness. The concentration of GelMA was increased 5, 10, 100, and 400 times to assess changes in GCX expression. In a control experiment, the same was done with gelatin. The results, as depicted in Figures 2B–D, showed that in GelMA samples, the normalized expression (MFI) of the whole GCX (WGA) nearly doubled and then reached a plateau in response to a 5- and 10-fold increase in GelMA concentration. Higher concentrations (100x and 400x) of GelMA coating led to cell detachment presumably due to the high concentration of methacrylate groups, potentially causing cell toxicity and detachment. In the control experiments with gelatin, increasing the concentration of gelatin did not significantly affect the expression of the whole GCX (WGA), as both the normalized expression (MFI) and thickness of the GCX remained similar across the samples (Supplementary Figure S5). The results shown in Figure 2, all taken together, suggest that the substrate material can indeed impact GCX expression. The use of gelatin coating and GelMA coating at moderately high concentrations may be better than fibronectin to accurately elicit natural GCX-mediated behavior of ECs for atherosclerosis-related studies.

### 3.5 The effect of substrate stiffness on GCX

To investigate the impact of substrate stiffness on GCX expression, hydrogels with stiffness values of 2.5, 5 and 10 kPa were employed. The hydrogels with stiffness values of 2.5 and 5 kPa hydrogels represented matrices with physiological stiffness, while the 10 kPa hydrogels represented pathological stiffness (Mahmoud et al., 2021b). Previous studies performed by our colleagues in the laboratory of Tarbell have shown that stiffness can disrupt the expression of glypican 1, a core protein of the GCX, as well as HS, a polysaccharide component of GCX, on fibronectin-coated polyacrylamide hydrogels (Mahmoud et al., 2021a). Our aim was to advance Tarbell’s work with continued study of GCX mechano-response to substrate stiffness, and we sought to do so by using non-swelling GelMA hydrogels in preparation for future research in which we will translate our GelMA platform to a flow chamber with geometry and flow parameter constraints.

As shown in Supplementary Figure S6, when cells were plated directly on the GelMA hydrogels as initially planned, it was found that cells did not consistently adhere well and exhibited delayed proliferation. Challenges were found to be particularly relevant to 2.5 kPa hydrogels. To overcome the potential adverse impact of GelMA on cellular adhesion and proliferation-to-confluence, the hydrogels were coated with 1x gelatin prior to cell seeding. To confirm that the gelatin could not change the GelMA mechanical properties detected by ECs, the gelatin was FITC-conjugated to visualize the gelatin coat thickness. After washing the hydrogel samples, it could be seen via FTIC that the gelatin is very thin on both soft and stiff hydrogels and, therefore, unable to affect the mechanical properties of GelMA. In addition, on 2.5 kPa hydrogels, cell attachment was monitored, and initial attachment to the gelatin-coated substrates was found to be superior compared to non-coated substrates. Cells exhibited sufficient attachment to 10 kPa hydrogels irrespective of coating (data not reported). Subsequently, the influence of uncoated GelMA *versus* gelatin-coated GelMA on GCX expression was investigated using WGA labeling, and no significant differences were observed between the coated and non-coated hydrogels. Therefore, the application of gelatin coating on GelMA hydrogel yielded fine-tuned cell culture optimization: gelatin coating of GelMA solely supported cell attachment to the softer gels and did not impact attachment on stiffer gels or GCX expression on hydrogels with different stiffnesses.

**FIGURE 3:**
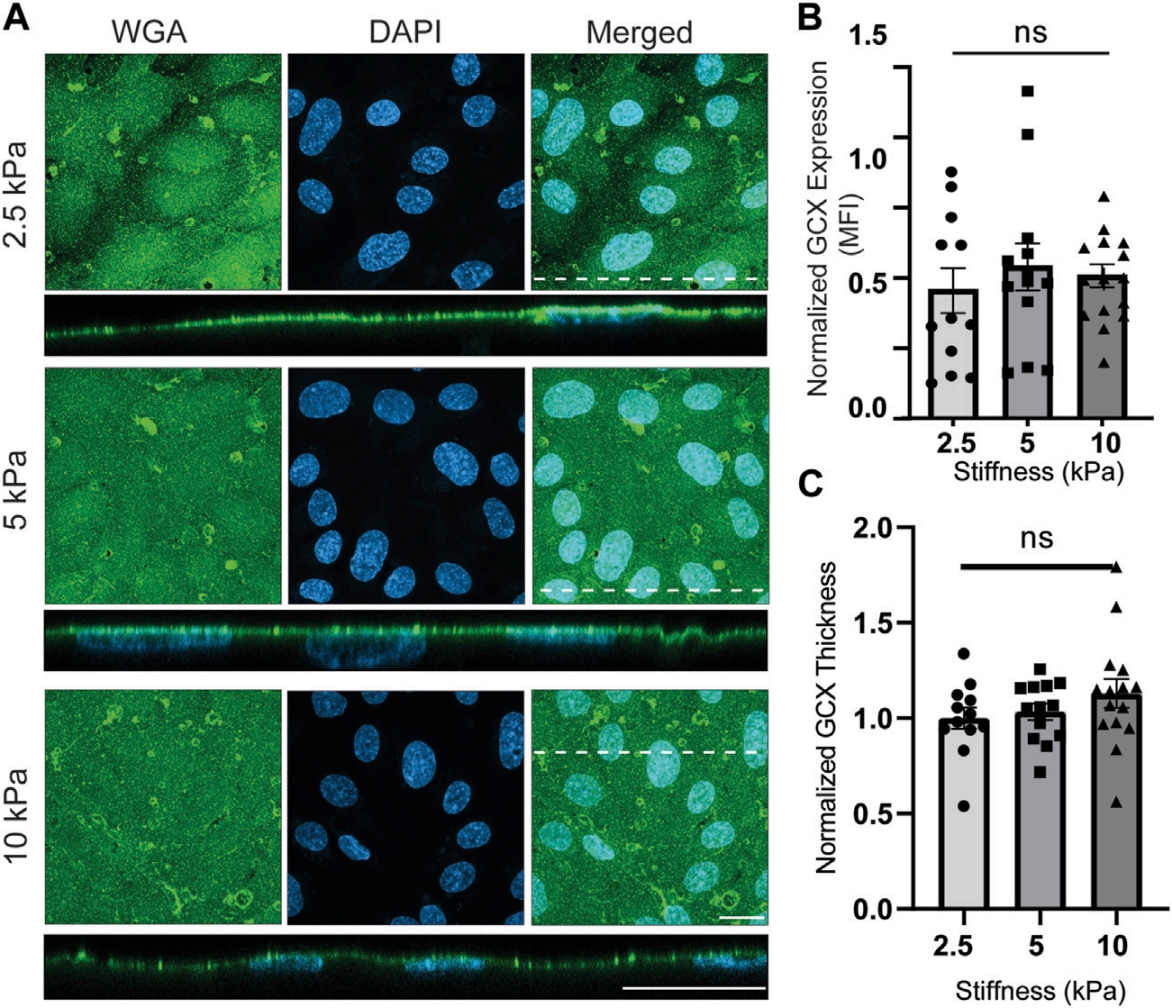
Physiological (2.5 and 5 kPa) and pathological (10 kPa) substrate stiffnesses do not alter overall GCX expression. **(A)** *En face* view of ECs showing the effect of GelMA hydrogel stiffness on apical GCX expression. Orthogonal view of the EC monolayer demonstrating apical GCX. Green is WGA, the marker of whole GCX, and blue is DAPI, the marker of EC nuclei. Dashed lines correspond to positions of orthogonal views (scale bars are 20 μm). **(B)** The MFI from the *en face* view of the microscopic images, showing that stiffness did not change whole GCX expression (N = 3, n = 3, mean ± SEM). **(C)** The thickness of the GCX measured from an orthogonal view did not show any significant difference due to variations in stiffness (N = 3, *n* = 3, mean ± SEM). The non-normalized data for this plot are as follows: 2.5 kPa hydrogel, 1.06 μm ± 0.103; 5 kPa hydrogel, 1.09 μm ± 0.074; 10 kPa, 1.19 μm ± 0.116.

For cells grown on gelatin-coated GelMA hydrogels, the effects of stiffnesses of 2.5 kPa, 5 kPa, *versus* 10 kPa were compared to each other. Qualitatively, WGA-labeled GCX was abundantly expressed. In *en face* images, occasional aggregation of the WGA lectin was seen. When scanning the *en face* images, the aggregates could be thought to be marking cell junctions. However, when scanning the cross-sectional images, the aggregates appeared to be randomly distributed and less obvious. Quantitative analysis of the *en face* images revealed that there were no significant differences in GCX (WGA) expression (MFI) detected when comparing GCX expression in 2.5 kPa, 5 kPa, *versus* 10 kPa conditions (Figures 3A, B). Similarly, the thickness measurements did not reveal any significant differences across samples with different stiffness values (Figure 3C). These observations prompted us to investigate the prevailing saccharide components of the GCX.

**FIGURE 4:**
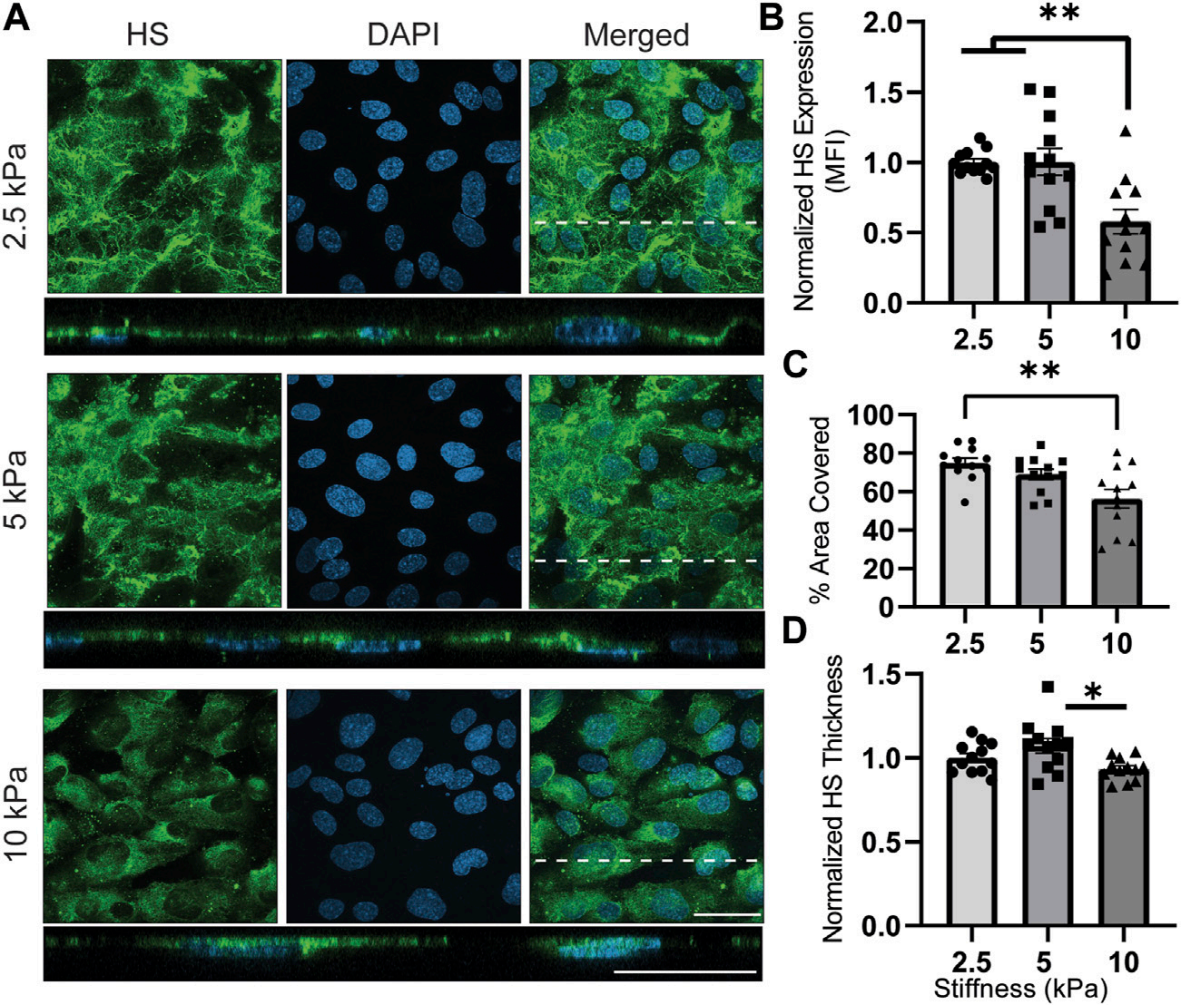
HS expression is downregulated on substrates with pathological (10 kPa) stiffnesses. **(A)** *En face* view showing the effect of GelMA hydrogel’s stiffness on HS expression. Orthogonal view of the EC monolayer demonstrating apical HS. Green is HS, a major saccharide-based component of GCX, and blue is DAPI, an EC nuclei marker. Dashed lines correspond to positions of orthogonal views (scale bars are 20 μm). **(B)** The MFI from the *en face* views of the microscopic images, showing increased expression of HS when observing the effect of moving from physiological stiffness to pathological stiffness (N = 4, n = 3, mean ± SEM, **p < 0.01). **(C)** The area covered with HS decreased on stiffer substrate with a significant different between 2.5 and 10 kPa hydrogels (N = 4, n = 3, mean ± SEM, **p < 0.01). **(D)** Thickness of the HS was measured showing a significant decrease from 5 kPa to 10 kPa (N = 4, *n* = 3, mean ± SEM, *p < 0.05). The non-normalized data for this plot are as follows: 2.5 kPa hydrogel, 1.72 μm ± 0.052; 5 kPa hydrogel, 1.84 μm ± 0.086; 10 kPa, 1.60 μm ± 0.039.

Figure 4A illustrates the presence of HS in response to varied stiffness on gelatin-coated GelMA. The ECs displayed lower HS expression on 10 kPa hydrogels, which resembled pathological stiffness, while no significant difference was observed between 2.5 and 5 kPa hydrogels, representing the physiological stiffness of blood vessels (Figure 4B). Similarly, the percentage of the EC-covered area occupied by HS decreased from 74% (2.5 kPa) and 69% (5 kPa) to 56% (10 kPa) when transitioning from physiological to pathological matrix stiffness (Figure 4C). Likewise, the thickness of the HS was decreased by increasing stiffness from physiological to pathological substrates (Figure 4D). These findings shown in Figure 4 align with the data published by Mahmoud et al. (Mahmoud et al., 2021b), which demonstrated HS downregulation with increasing stiffness. It is worth noting that in some areas, the orthogonal view revealed intracellular signals, despite the fact that the HS staining procedure did not include cell membrane permeabilization of the samples. Perhaps the long period of primary antibody incubation allowed the antibody ample time to passively diffuse through the cell membrane to the cytoplasm.

**FIGURE 5:**
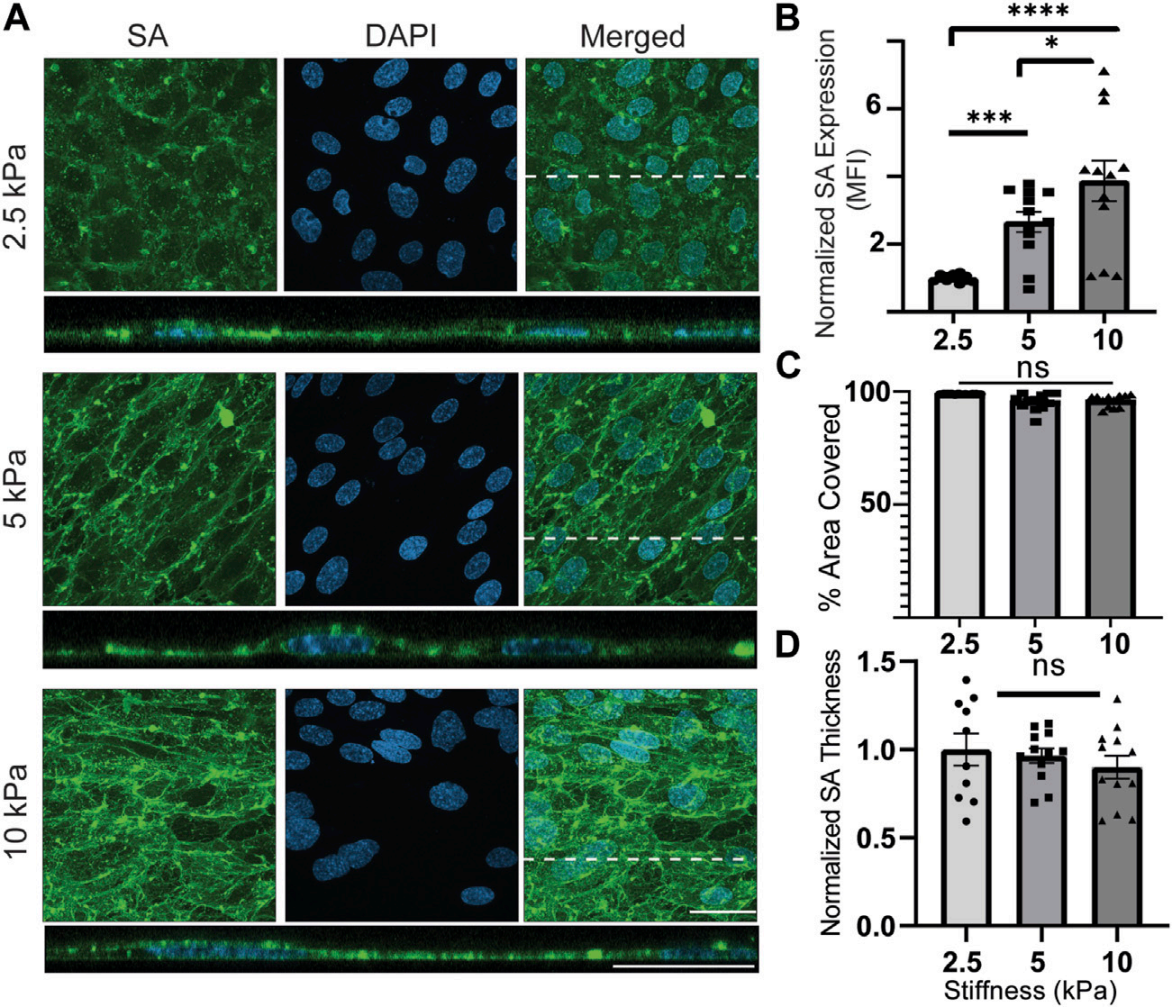
SA expression is upregulated on substrates with increased stiffnesses. **(A)** *En face* view showing the effect of GelMA hydrogel stiffness on SA expression. Orthogonal view of the EC monolayer demonstrating apical SA. Green is SA, and blue is the nuclei marker DAPI. Dashed lines correspond to positions of orthogonal views. (scale bars are 20 μm). **(B)** The MFI from the *en face* views of the microscopic images showed that increasing the stiffness increased expression of SA. (N = 4, n = 3, mean ± SEM, *p < 0.05, ***p < 0.001, ****p < 0.0001). **(C)** The area covered with SA was not affected by change of stiffness (N = 4, n = 3, mean ± SEM). **(D)** Thickness of the SA was also measured, showing no significant difference between stiffness conditions (N = 4, n = 3, mean ± SEM). The non-normalized data for this plot are as follows: 2.5 kPa hydrogel, 1.70 μm ± 0.162; 5 kPa hydrogel, 1.60 μm ± 0.082; 10 kPa, 1.48 μm ± 0.126.

Next, the presence of SA was examined (Figure 5A). Qualitatively, it could be observed in *en face* views that there was an apparent pattern of SA expression at cell junctions. However, the cross-sectional views did not provide further insight into any tendency for SA to mark cell junctions. Therefore, conclusions about cell junctional SA expression are to be approached with caution. The cross-sectional views pointed to something else that was of interest. The images showed occasionally that SA was present not only on the apical membrane of the cultured cells but also on the basal membrane. There was an attempt to quantify differential SA expression at the apical surface *versus* at the basal surface, as shown in Supplementary Figure S7. It appeared to be predominantly basal at 2.5 kPa and switch to predominantly apical at 10 kPa. However, these are cautious conclusions because the statistical analysis of this phenomenon could not be performed. Expression of basal GCX was rare and left a small sample size that could not be adequately processed with statistical tests. When examining the quantitative results, a correlation between substrate stiffness and SA expression was revealed, with cells exhibiting higher SA expression on stiffer substrates. Compared to 2.5 kPa hydrogels, normalized SA expression (MFI) increased by 2.5 times on 5 kPa hydrogels and 3.8 times on 10 kPa hydrogels (Figure 5B). However, there were no significant differences in area coverage or thickness of SA between the substrates with different stiffness levels. Although the thickness tended to decrease with increasing stiffness, the changes were not statistically significant (Figures 5C, D).

**FIGURE 6:**
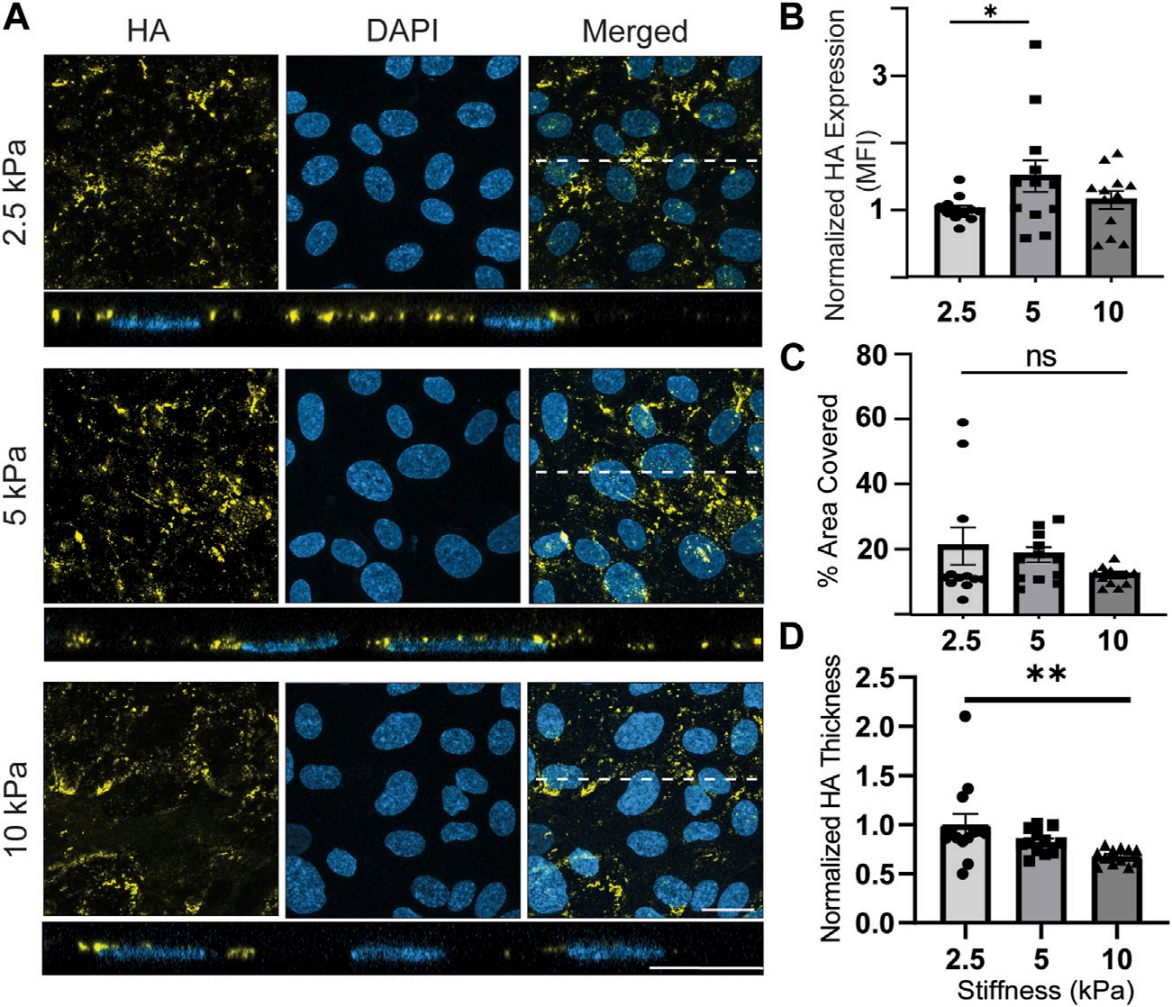
HA expression fluctuates while its thickness consistently decreases on stiffer substrates with 10 kPa stiffness (pathological conditions). **(A)** *En face* view showing the effect of GelMA hydrogel stiffness on HA expression. Orthogonal view of the EC monolayer demonstrating apical HA. HA expression is relatively low and false colored as yellow to make it more visible. Blue is DAPI, the EC nuclei marker. Dashed lines correspond to positions of orthogonal views (Scale bars are 20 μm). **(B)** The MFI from the *en face* views of the microscopic images, showing that stiffness increased expression of HA from 2.5 to 5 kPa and decreased HA again with further increasing stiffness to 10 kPa (N = 4, n = 3, mean ± SEM, *p < 0.05). **(C)** The area covered with HA was decreased by stiffness, but the changes were not significantly different (N = 4, n = 3, mean ± SEM). **(D)** Thickness of the HA decreased with increasing the stiffness of substrates with a statistically significant difference between the effects of 2.5 and 10 kPa substrates (N = 4, n = 3, mean ± SEM, *p < 0.05). The non-normalized data for this plot are as follows: 2.5 kPa hydrogel, 1.340 μm ± 0.149; 5 kPa hydrogel, 1.11 μm ± 0.047; 10 kPa, 0.91 μm ± 0.031.

The last component studied was HA (Figure 6A). Qualitatively, the cells appeared to express less HA than HS and SA. The was no apparent HA preference for cell junctions or basal expression of HA, which were interesting observations that were made for HS and SA. Quantitatively, normalized HA expression increased by a statistically significant 1.48-fold from 2.5 kPa to 5 kPa conditions but decreased back to approximately the same level as 2.5 kPa when stiffness increased from 5 to 10 kPa (Figure 6B). There was no significant difference when comparing the effects of 2.5 kPa to 10 kPa (Figure 6B). The percentage area of ECs covered with HA did not show a significant difference among the samples in different stiffness conditions, although a decreasing trend was observed on stiffer substrates (Figure 6C). Lastly, the thickness of HA layer exhibited a statistically significant decrease for samples exposed to highest stiffness conditions compared to samples exposed to lowest stiffness conditions (Figure 6D).

## 4 Discussion

The GCX is a crucial element of atherosclerosis, but its delicate and complex structure has made it difficult to study using traditional methods until recent technological advancements, which have made it more feasible (Hamrangsekachaee et al., 2022). Additionally, relatively recent studies have demonstrated the direct response of GCX to apical flow-derived shear stress, while many studies have overlooked the impact of the basal substrate on GCX features and function. To improve the accuracy of *in vitro* studies, it is essential to closely mimic the *in vivo* microenvironment, which includes a variety of mechanical, electrical, chemical, and other cues. As a first step toward improving model accuracy, we used a non-swelling hydrogel-based substrate with tunable properties. This substrate serves as a more physiologically relevant platform for incorporating mechanical stiffness of both healthy and diseased subendothelial matrices into our models. To the best of our knowledge, this is the first study to investigate the effect of substrate material chemistry on the holistic GCX. The results of our study will influence the substrate material choice for future studies. Furthermore, we expanded upon recently published findings on the effects of substrate matrix mechanical properties on GCX (Mahmoud et al., 2021b), adopting a holistic approach that provides insights into the responses of the entire GCX, as well as its subcomponent and the ECs, to different solid-derived forces. In future work, our substrate matrix model will be combined with different types of flow conditions to create a further optimized *in vitro* model for studying EC behavior and functionality.

### 4.1 Development of a hydrogel-based substrate with tunable properties to mimic healthy and diseased subendothelial matrix

To improve upon existing models for studying EC and GCX in the context of atherosclerosis, this work has developed a biologically, chemically, and mechanically relevant EC substrate. Several candidate substrate biomaterials such as polyethylene glycol, and tropoelastin were pilot tested, but it was determined that a non-swelling gelatin-based option is most suitable for the long-term goal of developing an optimized *in vitro* model introducing flow conditions for studying EC behavior. It is acknowledged that gelatin-based substrates have been extensively studied in recent decades, and their advantages in terms of sustainability, cost effectiveness, and biocompatibility have been established (Gómez-Guillén et al., 2011; Lee et al., 2015; Shirahama et al., 2016; Zhu et al., 2019). However, there is a unique aspect to our approach, as two well-established protocols were combined to enhance the reproducibility of the substrate batches while keeping the process simple (Kim et al., 2014; Zhu et al., 2019). Additionally, by modifying gelatin into GelMA (Figure 1), this study successfully achieved the goal of creating substrates with different stiffness levels while maintaining the shape and dimension of the substrate. This will be advantageous when integrating the substrate into a flow channel setting in the future, as it will prevent any unexpected flow disturbance resulting from hydrogel swelling.

### 4.2 Regulation of substrate components and their concentration

Since the mechanical properties of the hydrogel substrate are directly influenced by the concentration of GelMA, it was important to assess GCX expression based on both the composition and concentration of the protein used as the substrate, whether in coating or hydrogel form. In this assessment, we focused on the coating configuration. As a standard control, fibronectin was utilized and compared to gelatin and unpolymerized GelMA. Fibronectin has been widely used as a coating substrate in numerous research publications due to its high cell attachment properties (Stroka and Aranda-Espinoza, 2011; Yeh et al., 2012; Mahmoud et al., 2021a; Mahmoud et al., 2021b). Surprisingly, the overall expression of GCX on gelatin and GelMA was significantly different compared to fibronectin. Gelatin increased GCX expression, while GelMA initially decreased GCX expression. However, as the concentration of GelMA was increased, there was a significant increase in GCX expression, approaching the level of GCX expression on gelatin coated glasses (Figure 2). Interestingly, GCX expression did not change significantly in response to coating with higher concentrations of gelatin **(** Supplementary Figure S4 **)**. These observations suggest that under static conditions with the same substrate stiffness, GCX expression can be upregulated to a certain extent. The concentration of GelMA determines the stiffness of hydrogels, but it can be concluded that the number of cell-adhesive sequences in GelMA hydrogels are significantly above the threshold found in Figure 2. These findings also suggest that gelatin or GelMA (5x and 10x) could be considered as substitutes for fibronectin coating in atherosclerosis-related research using coated glass substrates, especially since it is known that fibronectin becomes a predominant constituent of the substrate matrix that ECs reside on under disease conditions. Elevated fibronectin levels have been shown to have adverse effects on the deposition, organization, and stability of other matrix adhesion proteins, as well as functional events such as endothelial permeability, cell adhesion, and cell proliferation (Sottile and Hocking, 2002; Chiang et al., 2009). Numerous studies have also demonstrated a correlation between high fibronectin levels and increased risk of atherosclerosis in human patients (Magnusson and Mosher, 1998; Tzanatos et al., 2009; Holm Nielsen et al., 2020). Therefore, it is important to limit or completely avoid experimental models that incorporate substrate protein that exacerbates atherosclerosis, as they may interfere with cell behavior and subsequently affect research data.

### 4.3 Increasing substrate stiffness differentially dysregulates individual polysaccharides without altering overall GCX

Numerous studies have demonstrated the impact of substrate stiffness on the behavior and function of ECs, including proliferation, migration, and even ECs stiffness. (Byfield et al., 2009; Lampi et al., 2017; Zhong et al., 2018; Bastounis et al., 2019; Krüger-Genge et al., 2021). However, only a few studies have specifically examined the influence of stiffness on GCX expression (Mahmoud et al., 2021a; Mahmoud et al., 2021b), and we aimed to contribute to this area of research. To understand the effect of substrate stiffness on GCX integrity, we first took a holistic approach, treating the GCX as a homogeneous saccharide coating that covers the cell surface and labeling it with WGA. Our results indicated that the intensity and thickness of expressed whole GCX did not change in response to substrate stiffness (Figure 3). This unexpected finding contradicted our initial hypothesis and differed from previous reports (Mahmoud et al., 2021a; Mahmoud et al., 2021b) that suggested a correlation between stiffness and GCX expression. Consequently, we shifted our focus to examining the alterations in three major polysaccharide components of the GCX.

As depicted in Figure 4, we initially examined the presence of HS. In healthy ECs, HS is continuously produced in the endoplasmic Golgi apparatus. It is the most predominant component among GAGs, comprising 60%–90% of GCX GAGs, and plays a crucial role in various functions. The physiological function of this GAG can be influenced by more than 4,000 different possible sulfation patterns (Reitsma et al., 2007; Spiess, 2017). Our findings indicate that HS is highly sensitive to substrate stiffness, which aligns with previous research (Mahmoud et al., 2021a; Mahmoud et al., 2021b). The expression (MFI; aggregate density) and thickness of HS decreased on the pathologically stiff surface (10 kPa), consistent with previous studies (Mahmoud et al., 2021a). The decrease in HS has significant adverse implications, as it is well-established that HS serves as a mechanotransducing factor involved in shear-induced NO production, cell motility, cell proliferation, and cell remodeling (Florian et al., 2003; Pahakis et al., 2007; Yao et al., 2007; Ebong et al., 2014). Although the present study did not involve flow-derived shear stress, our results suggested that a stiffer basement membrane could potentially interfere with flow-dependent cell functions, such as cell alignment and NO production, due to its associated lowering of HS expression. Additionally, HS acts as a mask for adhesion molecules like intercellular adhesion molecule 1 (ICAM-1) and vascular adhesion molecule 1 (VCAM-1) (Mulivor and Lipowsky, 2002). The decrease in the distribution and thickness of HS, as observed with increasing stiffness, implies that a stiff substrate may increase the likelihood of inflammatory cell adhesion via exposing ICAM-1 and VCAM-1 molecules on the EC surface. This conclusion is supported by previous reports showing that stiffer hydrogels promoted immune cell trans-endothelial migration (Huynh et al., 2011), and one possible reason for this observation could be the downregulation of HS on stiffer hydrogels. However, it is worth noting that while our findings regarding HS results and their implications align with prior published work, the disparity between the presence of HS and the overall GCX expression as measured by WGA was an unexpected finding.

The results obtained from examining SA provide an explanation for the disparity between the overall GCX and HS. As shown in Figure 5, although the thickness and coverage area of SA did not change significantly, its expression (MFI; aggregate density) was substantially upregulated on stiffer substrates. The information from Figure 5 clarifies the discrepancy between HS and the overall GCX, providing valuable insights and implications. One implication of increased SA, due to its repulsive negative charge (Wallach and Kamat, 1966), is that it enhances the maintenance of the GCX’s role as a permeability barrier. Another significant implication of increased SA, as it contains binding sites for recruiting immune cells and pathogens (Schauer, 2009; Chang and Nizet, 2014; Macauley et al., 2014; Mahajan and Pillai, 2016; Pearce and Läubli, 2016), is that it leads to enhanced inflammation. Considering these implications in conjunction with the HS results, it suggests that while the overall structure of the whole GCX (as indicated by WGA) is maintained, the relative concentrations of its individual components are altered. Furthermore, ECs respond to increasing stiffness conditions by adjusting the presence of HS and SA, not simply to counterbalance each other, but potentially to cooperate in adversely impacting EC functionality. In summary, the decrease in HS impairs mechanotransduction and exposes adhesion molecules, while the increase in SA recruits immune cells and pathogens, contributing to altered EC functionality under stiffer conditions.

To explore more about the effect of stiffness on the specific component of GCX, we examined the presence of HA (Figure 6). HA is a non-sulfated GAG that is part of the GCX and is bound to it through CD44 receptors on ECs. When HA is shed, it contributes to the loss of barrier functionality and can be associated with various disease conditions (Vlahu et al., 2012; Padberg et al., 2014; Tarbell et al., 2014). Previous studies have shown that the suppression of HA synthesis in mouse models leads to increased leukocyte adhesion and accelerated atherosclerosis (Nagy et al., 2010). Based on this well-established information about HA, we expected to observe a less distributed HA with lower thickness in EC cultures on pathologically stiff substrates. Although we observed a decrease in thickness, it was difficult to interpret due to the lack of meaningful correlations between the change of HA expression (measured by MFI; aggregate density) and the change in HA coverage of ECs. Our results suggest that stiffness has a negligible impact on promoting HA production. In future work, we plan to investigate whether the regulation of HA synthesis by stiffness can be induced by incorporating fluid flow as an additional factor in our model. By stimulating ECs with combined fluid flow and substrate stiffness forces, we can create a more complex but closer to accurate *in vitro* model, which would provide further insights into HA dynamics.

As proof of concept, this study demonstrates certain limitations that will be addressed in subsequent stages of the project. Firstly, the primary focus of the study centered on investigating the saccharide chains of the GCX in the absence of shear stress conditions. Shear stress is known to exert a critical influence on altering GCX. Consequently, the study did not extensively explore other vital components, including core proteins and the underlying mechanisms of GCX dysregulation. To establish a more comprehensive understanding of GCX, the forthcoming stage of this research will implement shear stress within the experimental setup. By doing so, the study will attain a more accurate representation of GCX responses to mechanical forces. The HUVECs used in this investigation do not fully represent the native endothelial cells of human arteries. However, their selection was based on a substantial body of data that allowed for the collection of comparable results and insights. In addition, the study did not include vascular smooth muscle cells (VSMCs), which are known to play a prominent role in atherosclerosis. The upcoming stages of the study will incorporate VSMCs to develop a more comprehensive multi-cell system that can be subjected to shear stress, thereby enhancing the accuracy of *in vivo* emulation. It is important to note that the hydrogels used in this study exhibited non-swelling behavior and possessed the capability to embed cells. Thus, the model can be exposed to shear stress, facilitating a more accurate representation of *in vivo* conditions.

## 5 Conclusion

In this work, we successfully modified gelatin through a straightforward method using methacrylate anhydrate, resulting in a reproducible biomaterial source for a substrate supporting the culture of ECs. Subsequent chemical, physical, and mechanical characterization confirmed the tunable nature of the hydrogel substrate. Notably, for the first time, gelatin and GelMA were compared to fibronectin, and it was found that gelatin at the same concentration of fibronectin could enhance GCX expression, while unpolymerized GelMA at higher concentrations also showed improved GCX expression. This finding provides a more cost-effective and sustainable alternative to fibronectin for endothelial studies. Furthermore, the impact of substrate chemistry and stiffness on GCX expression was evaluated. Interestingly, whole GCX expression was unchanged when ECs were cultured on physiologically soft *versus* pathologically stiff hydrogels. However, the individual GCX polysaccharides showed dysregulation in response to the mechanics of the hydrogels, with HS being downregulated and SA upregulated with increasing substrate stiffness. There was no strong correlation found between HA dysregulation and stiffness. The different responses of the various GCX components likely have direct or indirect effects on EC functions, given the diverse role of each component. It is important to note that the study did not assess EC functions beyond GCX production, which is the subject of a separate ongoing investigation where the EC substrate presented in this study is combined with fluid flow to create a robust *in vitro* model. The findings of this study provide new insights into how ECs respond to external forces and adapt their GCX to regulate EC function under various mechanical conditions. Further studies are needed to elucidate the mechanisms involved in GCX adaptation at the cell-matrix interface, such as those involving focal adhesions and integrins. Additionally, future work in a more complex *in vitro* system that incorporates both solid- and flow-derived forces will improve our understanding of the GCX response to its mechanical environment and the underlying mechanisms. In conclusion, the data and new knowledge presented in this study serve as a solid foundation for further exploration in the field.

## Data Availability

The raw data supporting the conclusion of this article will be made available by the authors upon request.
