# Supplementary material for: Endothelial glycocalyx sensitivity to chemical and mechanical sub-endothelial substrate properties
Source: Front Bioeng Biotechnol. 2023 Oct 30;11:1250348. doi: 10.3389/fbioe.2023.1250348 (PMC10643223; doi:10.3389/fbioe.2023.1250348)
Supplement: Supplementary file 1 [file DataSheet1.PDF]

Supplementary Material

Endothelial Glycocalyx Sensitivity to Chemical and Mechanical Sub-Endothelial Substrate Properties

Mohammad Hamrangsekachaee[1], Ke Wen[1], Narges Yazdani[2], Rebecca Willits[1,2], Sidi A. Bencherif[1,2,3,4*], and Eno E. Ebong[1,2,5,*]

*Correspondence:

Eno E. Ebong, Ph.D., Department of Chemical Engineering, Northeastern University, 805 Columbus Avenue, 221 Interdisciplinary Science & Engineering Complex, Boston, MA 02120

Phone: 617-373- 8744, Email: e.ebong@northeastern.edu

Sidi A. Bencherif, Ph.D., Department of Chemical Engineering, Northeastern University, 360 Huntington Avenue, 336 Mugar Life Sciences Building, Boston, MA 02115

Phone: 617-373-7103, Email: s.bencherif@northeastern.edu

Table S1: The parameters used for confocal microscopy.

Marker	Microscope	Laser power (%)	Detector Gain (V)	Z-stack Distance (μm)
WGA (On glass slides)	Zeiss LSM 800	0.4	625	0.19
WGA (Hydrogel)	Zeiss LSM 800	0.4	625	0.19
HS	Zeiss LSM 710	2.4	750	0.38
SA*	Zeiss LSM 710	2.5	800	0.38
	Zeiss LSM 800	0.2	550	0.38
HA	Zeiss LSM 800	0.1	500	0.38

*: Two microscopes were utilized for SA imaging, and the data was normalized to 2.5 kPa samples obtained from the same microscopes to remove the variation among microscopes.

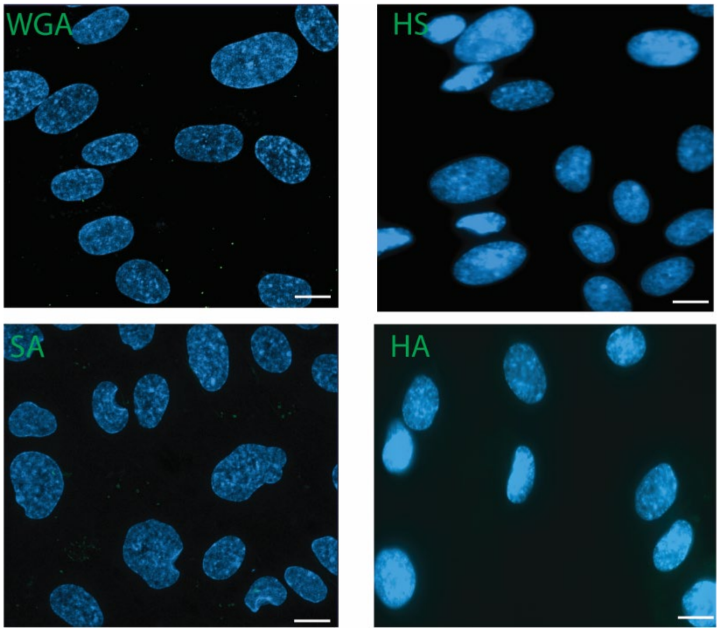

Figure S1: Negative controls to assess the lectin, binding protein, and antibody specificities and extract non-specific signal data. Negative controls were defined as samples in which lectins, binding proteins, or antibodies were omitted while all other histology steps were performed. The performance of negative control experiments resulted in greatly reduced fluorescence, as shown in this figure. These results confirm that the staining observed in the HUVEC is not artifactual. Omission of biotinylated WGA lectin, biotinylated elderberry bark lectin, clone F58-10E4 antibody against HS, and HA binding protein was also used to generate fluorescent signal data of the non-specific signals. The non-specific signal data were subtracted from the data collected for quantification of intensity, thickness, and area coverage (scale bar is 10µm).

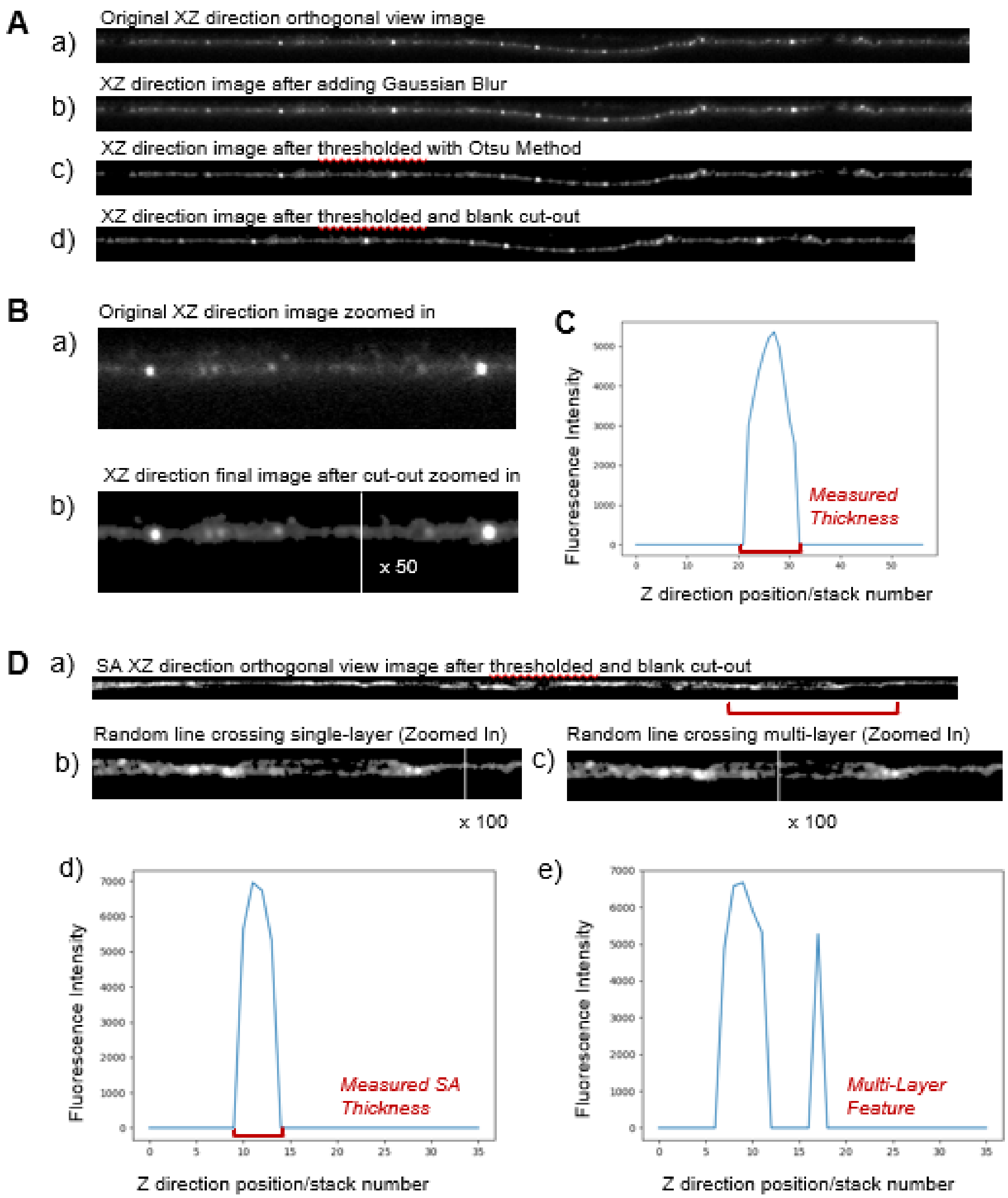

Figure S2: Step-by-step illustration of the thickness measurement method. (A) a) XZ direction orthogonal view image of WGA-stained sample cultured on gelatin-coated glass; b) XZ direction image after adding gaussian blur; c) XZ direction image gaussian blur was followed by Otsu threshold

processing such that any pixel in whose intensity lower below the threshold (found with automated method) was turned to 0 and shown as black/blank; d) XZ direction image after next cutting out areas containing only blank pixels to obtain the processed Region of Interest (ROI). **(B)** a) Zoomed in image of above image that is denoted as A.a., where area of GCX is unclear with ambiguous edges; b) Zoomed in image of above image that is denoted as A.b., where ROI is clearly defined after processing. **(C)** An example intensity profile of a vertical line drawn at a randomly on the ROI (see the white line in the image that is denoted as A.d.). The GCX thickness was measured as the length of non-blank pixels (intensity greater than 0). For one sample image, 50 similar random lines were drawn, and GCX thickness was averaged over 50 measured thickness values. **(D)** a) XZ direction orthogonal view image of SA showed occasional encapsulation of ECs by SA, and from this image areas that only contain blank pixels in the Z direction was cut out to obtain the processed Region of Interest (ROI); b) a randomly drawn line crossing single-layer feature (shown in white) on the ROI; c) a randomly drawn line crossing multi-layer feature on the ROI; d) an example intensity profile of the randomly drawn line crossing the single-layer feature shown in the image denoted as D.b. shows a single peak; e) intensity profile of a randomly drawn line crossing the multi-layer feature shown in the image denoted as D.c. shows two peaks that can be analyzed to measure the apical and basal SA thickness separately. Subsequently, the apical thickness was documented alongside the thickness of the single layer. All images shown here have been tuned after being processed with the Python program for brighter presentation.

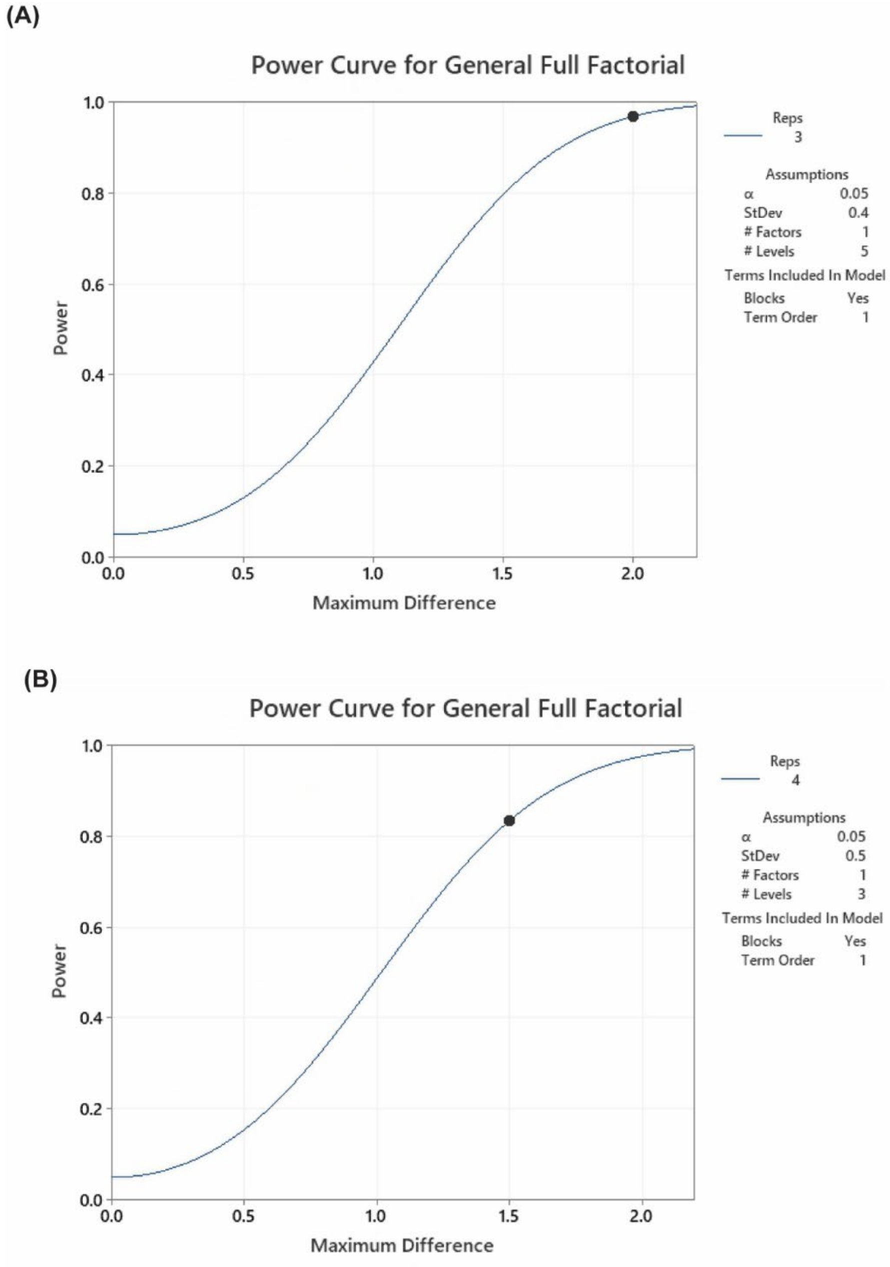

Figure S3: Power analysis based on preliminary data confirmed the statistical rigor of the experimental design. (A) The power curve for GCX expression in response to substrate coating chemistry (specific composition and component concentration) when maximum difference and standard deviation are 2 and 0.4, respectively, shows that with three replicates, the power is above 0.9 for a significance level of 0.05. (B) The power curve for GCX components in response to substrate

stiffness when maximum difference and standard deviation are 1 and 0.5, respectively, shows that with four replicates, the power is above 0.8 for a significance level of 0.05.

(A)

Hydrogel	Gelation time (s)
3% GelMA	132
4% GelMA	78
8% GelMA	48

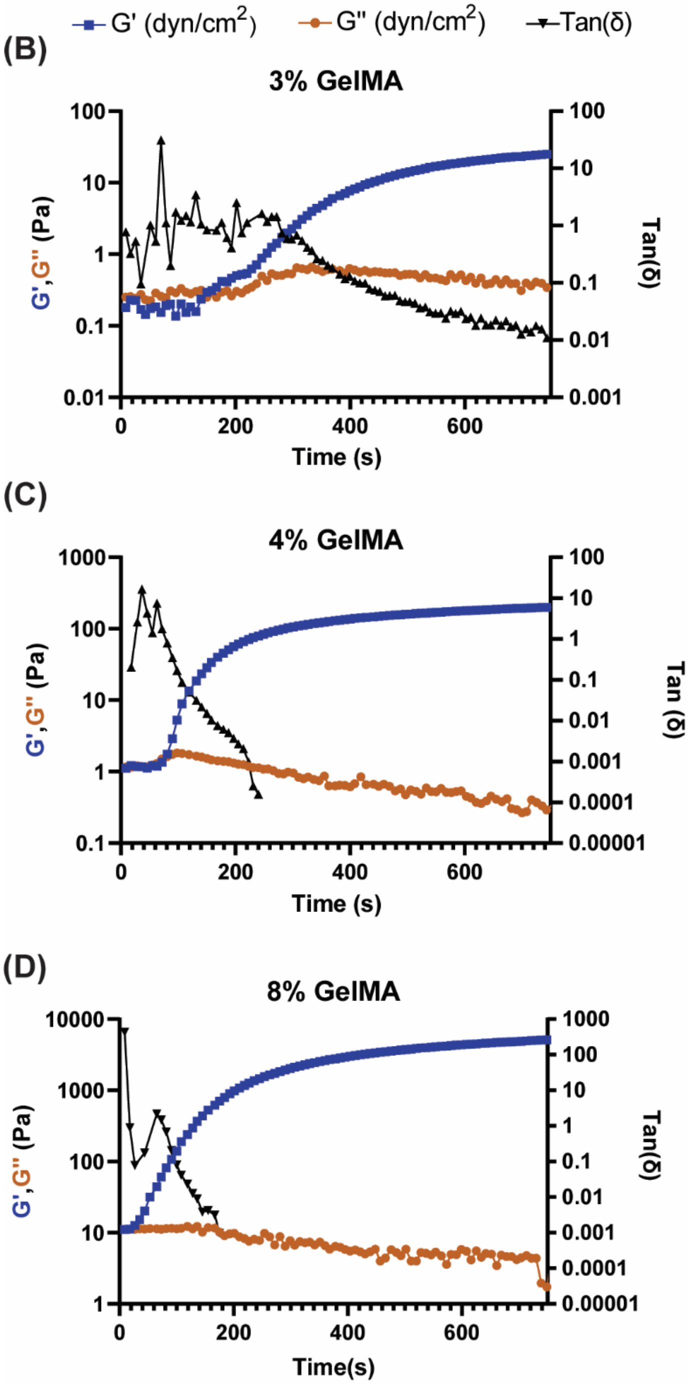

Figure S4: Gelation time and rheology of the hydrogels. **(A)** Increased concentration of GelMA caused the gelation point to occur more rapidly. This is due to the higher density of the reactant methacrylate groups. A The polymerization process initiates with the addition of TEMED and APS to the polymer solution. A precise 30-second interval was measured between the mixing of initiators with the polymer solution and the commencement of the test. This duration was taken into account when determining the gelation times presented in the table. **(B)**, **(C)**, and **(D)** The storage modulus (G') and loss modulus (G") increased with distinct slopes, while G' approached a plateau state, G" decreased further. The significant difference between G' and G" indicated that the hydrogels exhibited predominantly elastic behavior and were designed to resemble a more elastic material gel. It is crucial to emphasize that rheometry was exclusively employed to determine the gelation time of the samples. Any disparities observed between the rheometric results following conversion and the young moduli obtained through compression testing could potentially be attributed to variations in the experimental conditions.

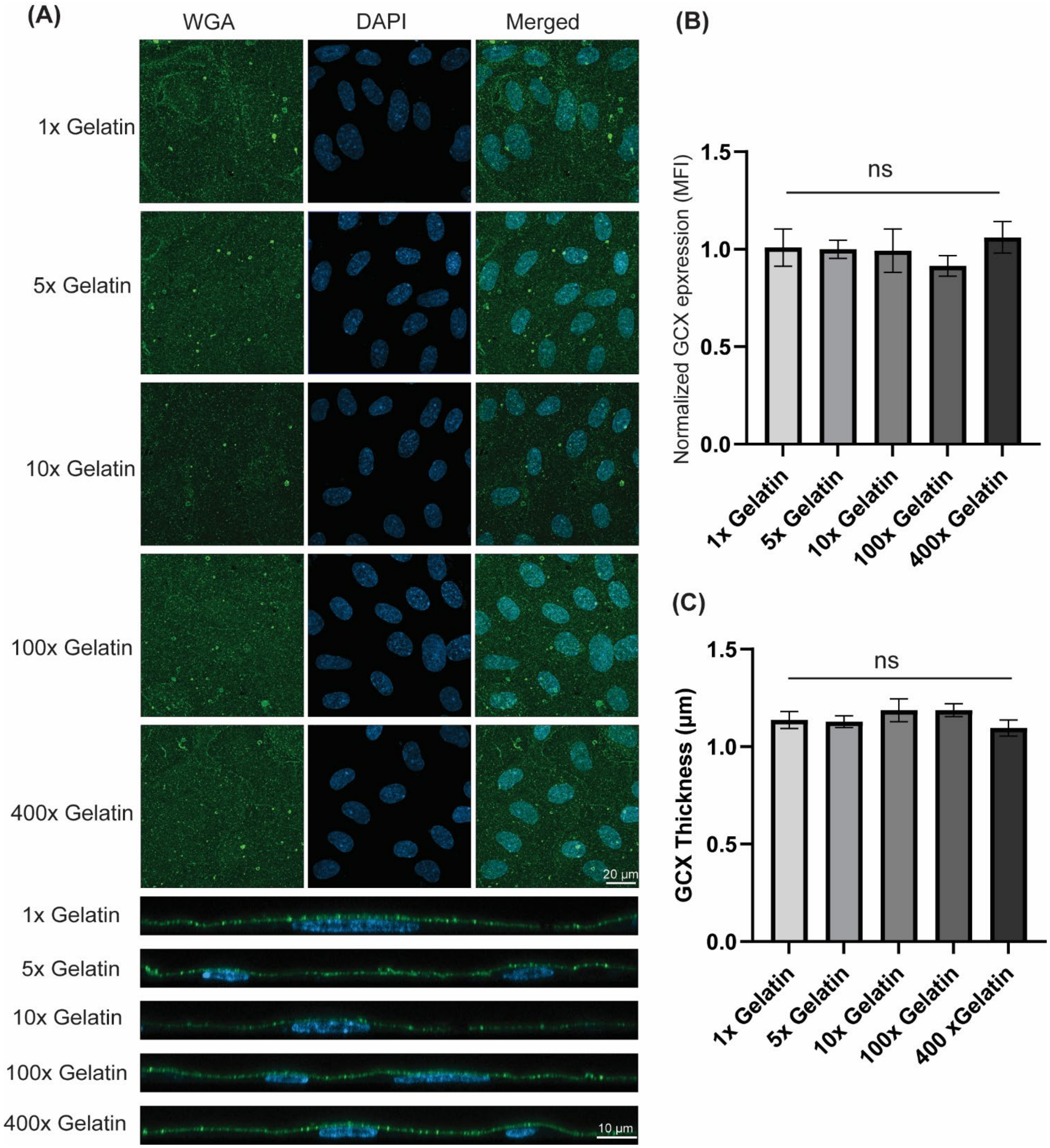

Figure S5: The concentration of gelatin do not dysregulate holistic GCX expression. **(A)** En face views showing the effect of substrate material on apical GCX expression. Orthogonal views of the EC monolayer demonstrating apical GCX, created from stacking images. Green is WGA, the marker of

whole GCX, and blue is DAPI, the EC nuclei marker (scale bar is 20µm in en face view and scale bar is 10µm in orthogonal view). **(B)** The MFI values from the en face views of the microscopic images, showing that increasing the concentration of gelatin did not affect the GCX expression (N=3, n=3, mean ± SEM). **(C)** Thicknesses of the GCX were measured from the orthogonal views and did not show any significant differences between the gelatin concentrations (N=3, n=3, mean ± SEM).

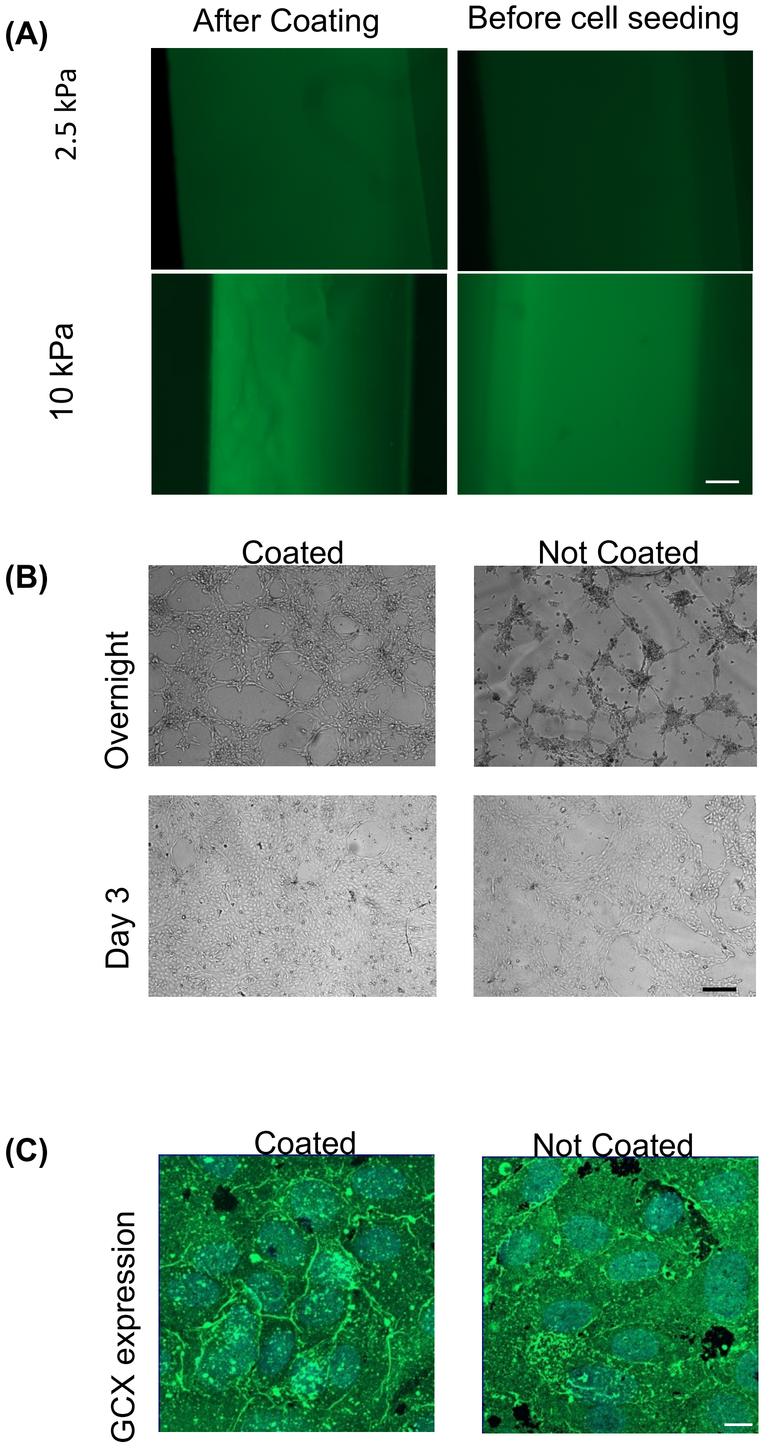
(A)
After Coating
Before cell seeding
2.5 kPa
10 kPa
(B)
Coated
Not Coated
Overnight
Day 3
(C)
Coated
Not Coated
GCX expression

Figure S6: Coating hydrogels with gelatin improve the initial cell attachment on 2.5 kPa hydrogels and do not alter GCX expression. (A) FITC-conjugated gelatin solution (1x= 60 µg/mL) was used to coat the hydrogels. Microscopic photos were taken to assess the thickness of the gelatin coat on hydrogels after coating and just before cell seeding. The photos confirm that the gelatin coat was not substantially thick on the hydrogel at the time of cell seeding because the gelatin diffused into the hydrogel (scale bar is 200µm). **(B)** Microscopic image of EC culture on hydrogels of 2.5 kPa stiffness demonstrating gelatin coating improved the initial cells attachment (scale bar is 200µm). **(C)** En face view showing that coating the hydrogels with gelatin did not impact apical GCX expression. Green is WGA, the holistic marker of GCX, and blue is DAPI, the EC nuclei marker (scale bar is 20µm).

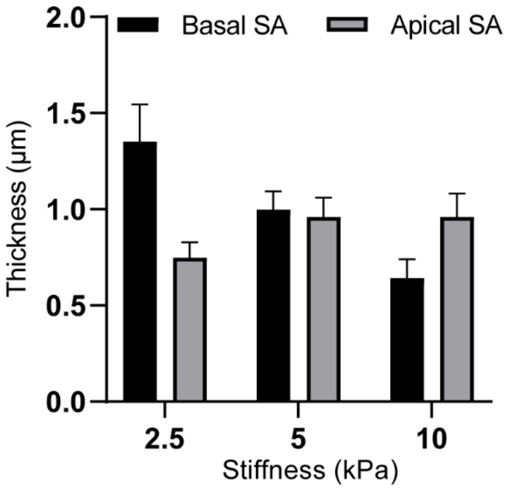

Figure S7: The cross-sectional views of SA for cells grown on gelatin-coated GelMA occasionally showed that SA is present not only on the apical membrane of the cultured cells but also on the basal membrane, with increase in the substrate's stiffness leading to an increase in the apical thickness of SA compared to basal thickness of SA. Preliminary quantification showed that on the 2.5 kPa substrate, SA was primarily expressed on the basal side of the cells. As substrate stiffness increased, more apical SA was expressed, and the apical SA thickness exceeded that of the basal SA on the 10 kPa substrates. Note that, for multi-layer SA expression measurements, the basal and apical layers were individually analyzed using a different algorithm than what is described in the main manuscript. The prior-mentioned method was used, but with the application of 100 drawn vertical lines. The features of basal and apical layers were identified based on the intensity profile plotted along the vertical line. Multi-layer (both basal and apical layer) expression was determined in cases where two significantly separatable peaks were detected on the profile (see Figure S2). (If only one peak was found, the expression was categorized as a single layer.) The measured apical thickness and basal thickness values were averaged for the sample. To occurrence of basal SA was rare and provided insufficient data points for full analysis of multilayer SA expression. Therefore, the data could not undergo statistical analysis and is merely described by mean ± SEM.
